# Phase segregation in mixed-halide perovskites affects charge-carrier dynamics while preserving mobility

**DOI:** 10.1038/s41467-021-26930-4

**Published:** 2021-11-29

**Authors:** Silvia G. Motti, Jay B. Patel, Robert D. J. Oliver, Henry J. Snaith, Michael B. Johnston, Laura M. Herz

**Affiliations:** 1 Department of Physics, University of Oxford, Clarendon Laboratory, Parks Road, Oxford, OX1 3PU United Kingdom; 2 TUM Institute for Advanced Study, Technische Universität München, Lichtenbergstr. 2a, 85748 Garching bei München, Germany

**Keywords:** Solar cells, Solar cells

## Abstract

Mixed halide perovskites can provide optimal bandgaps for tandem solar cells which are key to improved cost-efficiencies, but can still suffer from detrimental illumination-induced phase segregation. Here we employ optical-pump terahertz-probe spectroscopy to investigate the impact of halide segregation on the charge-carrier dynamics and transport properties of mixed halide perovskite films. We reveal that, surprisingly, halide segregation results in negligible impact to the THz charge-carrier mobilities, and that charge carriers within the I-rich phase are not strongly localised. We further demonstrate enhanced lattice anharmonicity in the segregated I-rich domains, which is likely to support ionic migration. These phonon anharmonicity effects also serve as evidence of a remarkably fast, picosecond charge funnelling into the narrow-bandgap I-rich domains. Our analysis demonstrates how minimal structural transformations during phase segregation have a dramatic effect on the charge-carrier dynamics as a result of charge funnelling. We suggest that because such enhanced recombination is radiative, performance losses may be mitigated by deployment of careful light management strategies in solar cells.

## Abstract (web summary)

Phase segregation in mixed halide perovskite is known to alter the optoelectronic properties, but how it affects charge carriers is not clear. Here, the authors use THz spectroscopy to reveal that high carrier mobilities are well preserved, while recombination dynamics is affected by charge funnelling upon segregation.

## Introduction

Lead halide perovskite semiconductors have experienced extraordinary progress over the last few years, taking the spotlight as the most promising novel materials for photovoltaics, with devices now reaching record efficiencies above 25%^1^. Following the optimisation of efficiencies of research-scale single-junction devices, more effort has been directed towards solving practical issues that prevent perovskite solar cells from reaching large-scale commercialisation. One such issue is the inadequate stability of some of these materials over long timescales, largely related to their high ionic mobility. Another important challenge is the optimisation of devices based on alternative compositions beyond the traditional lead triiodide perovskites, such as mixed-metal and mixed-halide compositions. Integrating these materials in tandem devices minimises losses associated with higher energy photons, significantly improving cost-effectiveness. For instance, mixed halide perovskites APb(Br_*x*_I_1−*x*_)_3_ (where A is a monovalent cation) can be fabricated in a wide range of bandgaps by varying the Br/I ratio. However, it is widely known that such materials undergo halide segregation when photoexcited, resulting in a heterogeneous landscape of compositions and bandgaps^2,3^. While much effort has been dedicated towards understanding and preventing the mechanisms driving halide segregation^3–8^, the actual implications of such transformations for the semiconductor performance are not yet fully understood. Recently, Mahesh et al.^9^ investigated the impact of halide segregation on the open-circuit voltage (V_OC_) of solar cells and found that V_OC_ losses in tandem-relevant materials as a result of non-radiative recombination dominate over the losses associated with the formation of phase-segregated domains. Caprioglio et al.^10^ have also shown that the increased radiative efficiency of the phase-segregated material can counterbalance to a certain extent the voltage losses associated with the narrow-gap minority phases. These findings raise the intriguing question of whether present attempts to fully suppress halide segregation should instead focus on achieving a better understanding and control over recombination dynamics in the segregated material.

In this work we address these aims by employing a combination of photoluminescence (PL) and optical-pump terahertz-probe (OPTP) spectroscopy to investigate the impact of phase segregation on the charge-carrier dynamics and transport properties in mixed halide perovskite films. We analyse the appearance of phonon-related features in the frequency-resolved photoconductivity, which derive from phonon anharmonicity, indicating ionic displacement is facilitated in the phase-segregated material. Such features are also demonstrated to serve as a novel and interesting tool for tracking the charge-carrier dynamics in heterogeneous perovskite films. We report the THz effective charge-carrier mobilities before and after phase segregation and show that charge carriers within the segregated domains maintain high mobility and are thus not strongly localised. However, we also reveal how charge funnelling into I-rich domains results in a dramatic acceleration of the charge-carrier radiative recombination in phase-segregated mixed halide perovskite. Although increased radiative efficiency is usually sought for optimisation of photovoltaic materials, in this case, the high radiative rates in the phase-segregated perovskite can translate into excess losses in mixed halide solar cells. Our findings thus indicate that the detrimental effects of halide segregation derive mostly from accelerated charge-carrier recombination, with local efficient charge transport largely being preserved.

## Results and discussion

### Charge transport

**Fig. 1:**
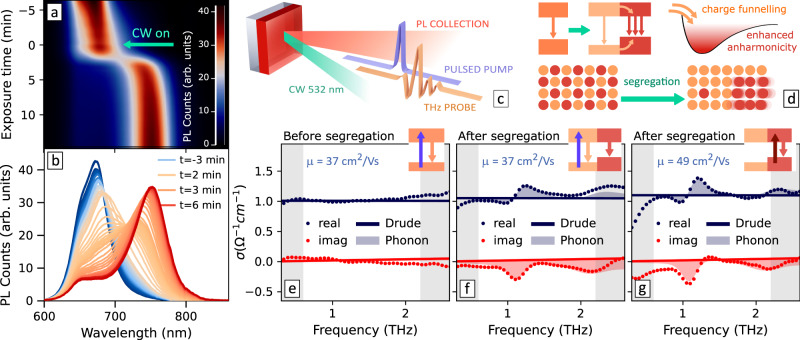
Phase segregation of mixed halide perovskites monitored by optical-pump terahertz-probe spectroscopy. **a**, **b** PL spectrum evolution over time under 400 nm pulsed illumination, collected in situ during the OPTP experiment, as illustrated in (**c**). Exposure to the CW laser starts at *t* = 0. **d** Diagram illustrating the effect of halide segregation with enhanced anharmonicity in the I-rich phase. The band diagram is for illustration purposes and does not mean to provide an accurate description of band alignment. **e**–**g** THz photoconductivity spectra of the MAPb(I_0.5_Br_0.5_)_3_ thin film at 5 ps after photoexcitation with 400 nm pump before (**e**) and after (**f**) phase segregation, and with 720 nm photoexcitation directly into the segregated I-rich phase (**g**).

To probe the effect of halide segregation on charge-carrier mobilities, we employed OPTP photoconductivity spectroscopy, where pulsed photoexcitation generates a transient population of charge carriers that is probed by the THz pulse. The changes to the THz transmission (Δ*T*/*T* ) induced by the pulsed photoexcitation are proportional to the photoinduced conductivity, providing insight into charge-carrier mobilities and populations^11–13^. While the value of the photoconductivity immediately after photoexcitation allows calculation of the charge-carrier mobility, charge-carrier recombination dynamics over the fs to ps timescales are revealed by varying the time delay *t*_pump_ between the laser photoexcitation and THz probe pulses. The extent and timescales of phase segregation in mixed halide perovskites are subject to several experimental factors, such as temperature, illumination intensity, and photoexcitation wavelength.^3,14,15^ Conveniently, mixed halide perovskite films are significantly more stable against halide segregation under pulsed illumination compared to continuous-wave (CW).^7,16^ Under pulsed illumination, halide segregation is expected to occur to a smaller degree and over much longer timescales.^14^ This feature allows us to employ pulsed light during the OPTP measurements to study the optoelectronic properties of mixed halide perovskites without tampering with the material structure. By adding a sufficient intensity of background CW light to induce halide demixing, we are able to compare the photoconductivity recorded before, during, and after the phase segregation, given that steady-state illumination does not affect the modulated OPTP signal. Furthermore, the charge-carrier density generated by the CW laser is orders of magnitude lower than that of the pulsed photoexcitation and does not significantly impact the analysis of recombination dynamics (see Supplementary Note 4). We have also confirmed that the CW illumination caused no irreversible degradation or other photoinduced transformations to the perovskite film (see Supplementary Note 5, showing the reversibility of the effects reported in this work). To monitor the extent of halide segregation, the steady-state PL of the samples was collected in situ during the OPTP experiments and recorded with a fiber-coupled spectrometer. The PL of a MAPb(I_0.5_Br_0.5_)_3_ film under 400 nm pulsed photoexcitation at fluence 11 μJ/cm^2^ is shown in Fig. 1a, b. A 532 nm CW laser beam was used to drive the phase segregation with 100 W/cm^2^ illumination intensity. Exposure to the CW light (starting at ${t}_{{{{{{{{\rm{exposure}}}}}}}}}=0$, indicated by a green arrow in Fig. 1a, b) results in halide segregation, suppressing the mixed-phase PL and creating a low-energy PL peak associated with the I-rich domains.

We used the photoconductivity amplitude at *t*_pump_ close to zero (i.e., before charge-carrier recombination takes place) to extract the effective sum mobilities μ of electrons and holes excited into the mixed-halide phase with 400-nm light.^11,13^ A value of *μ* = 37.3 ± 2.7 cm^2^/(Vs) was obtained for the mixed-halide phase before halide segregation had been induced. The film was then exposed to the segregation-driving CW illumination and the measurements were repeated after stabilisation of the PL spectral changes, resulting in *μ* = 37.2 ± 0.6 cm^2^/(Vs) . Such similar *μ* values of charge carriers immediately after photoexcitation are consistent with halide segregation occurring only in a small portion of the film despite drastic changes in PL lineshape^2,9,17^, which results in the minimal impact of halide demixing on the charge transport properties of the majority phase. This finding agrees with our recent observations in a parallel study carried out on identical films, in which XRD diffraction showed good preservation of material crystallinity upon halide segregation^17^. To understand the impact of heterogeneities on the transport properties, we tuned the OPTP photoexcitation wavelength to 720 nm. This low-energy pump falls below the bandgap of the majority mixed-halide phase and results in negligible Δ*T*/*T* signal before phase segregation had been induced (Supplementary Fig. 22), but can generate charge carriers directly into the narrow-bandgap segregated I-rich domains. We note, however, that the accurate determination of the absorption coefficients of the phase-segregated film at 720 nm is challenging, leading to uncertainties in the calculated mobilities. Our adopted calculation method results in *μ* = 49 cm^2^/(Vs) for charge carriers photogenerated by the 720 nm pump. Based on our evaluation of uncertainties, we also establish lower and upper boundaries of *μ*_low_ = 35 cm^2^/(Vs) to *μ*_high_ = 66 cm^2^/(Vs) (see Supplementary Note 6), from which we can confidently place the charge-carrier mobilities in the phase-segregated I-rich domains at values very close to or higher than those of the majority wide-bandgap phase.

The higher charge-carrier mobilities in the I-rich phase can be explained by the higher intrinsic mobilities in lead iodide perovskites compared to their bromide counterparts, owing to reduced Fröhlich coupling^18^. It is nonetheless remarkable to find such high mobilities in the heterogeneous landscape of the phase-segregated semiconductor, which suggests that such local domains add surprisingly few additional scattering pathways. We therefore further investigated how the charge transport mechanisms are impacted by halide segregation by measuring the frequency-resolved OPTP photoconductivity spectra. Figures 1e, f show the spectra at time delay *t*_pump_ = 5 ps after 400 nm photoexcitation before (${t}_{{{{{{{{\rm{exposure}}}}}}}}} \, < \,0$) and after (${t}_{{{{{{{{\rm{exposure}}}}}}}}} \, > \,0$) phase segregation. We also acquired the spectra with direct photoexcitation of the I-rich domains in the phase-segregated film at 720 nm (Fig. 1g). We observe in the three cases that the spectra show imaginary photoconductivity close to zero while the real part is relatively flat (aside from phonon modulations discussed further below). This is consistent with a Drude behavior, in which a charge accelerated under the THz electric field undergoes random scattering events at a rate that is inversely proportional to its mobility. For the alternative scenario of charges being confined within small domains, one would instead expect significant backward scattering, which would result in real photoconductivity spectra bending towards zero at low frequencies, accompanied by a negative imaginary part^19–23^. We have recently reported such behavior in perovskite nanocrystals between 6–10 nm in size^24^. While the size of I-rich domains in segregated mixed halide films has been reported to grow to similar scales^8,10,25^, even upon direct photoexcitation of these domains (Fig. 1g) our THz spectra demonstrate an absence of strong carrier localisation. Both our analysis of the photoconductivity spectra and the high charge-carrier mobilities measured thus demonstrate that local charge transport in both the dominant mixed-halide and the I-rich segregated phase is remarkably efficient.

It is important to note that the high frequency of the THz probe results in measured mobilities and photoconductivity spectra corresponding to short-range carrier motion, which does not necessarily translate into long-range transport^13,24,26^. The extraction of photogenerated charge carriers in a solar cell device also depends on the presence of sufficient long-range percolation pathways within the absorber for charge carriers to reach extraction layers. However, recent measurements of photocurrent spectra for mixed-halide solar cells have demonstrated that direct excitation of I-rich domains can indeed lead to photocurrent^9,15^. Thus percolation pathways within the I-rich phase of halide-segregated perovskites appear to exist, which together with our findings of high charge-carrier mobilities and Drude-like photoconductivity (Fig. 1g), strongly suggests that charge transport does not have a major impact on suppressing the performance of mixed-halide perovskite solar cells following halide segregation. This finding is in agreement with the minimal impact of halide segregation observed in the short-circuit current densities of mixed halide photovoltaic devices^9^.

### Phonon anharmonicity

We then turn our attention to another striking feature of the photoconductivity spectra in Fig. 1e–g, caused by phonon anharmonicity. These spectra reveal modulations superimposed onto the Drude response, seen as a peak derivative shape over the real part of the spectra and an associated negative peak over the imaginary part. These features are centered approximately at the frequencies of the two optical phonon modes of the lead halide sub-lattice^27–29^ evident in the THz transmission in the dark at ~1 and 2 THz (see Supplementary Fig. 1). The presence of such phonon modes is known to result in a series of distortions in the THz photoconductivity spectra, including artifacts^30,31^. The distinctive derivative shape on the real part of the spectrum evident in Fig. 1f, g cannot be explained by such artifacts, which have been corrected for (see details in Supplementary Note 1). Instead, these features are consistent with a transformation of the phonon modes in the presence of photoexcited charge carriers, associated with phonon anharmonicity^32,33^ or generally any form of structural distortions of the lattice in the photoexcited state^34–37^. As proposed by Zhao et al.^38^ such transformations can be described by a shift of the center frequency of the phonon modes in the photoexcited state (Δ*ω*) away from the equilibrium resonance frequency (i.e., in dark, *ω*_0_) combined with variations of the oscillator strength and background dielectric function. We applied a similar analysis to model the spectra measured for the mixed halide films (see Supplementary Note 1 for details). Figure 1e–g shows the OPTP spectra, fitted as a combination of a Drude response (plotted as solid lines) with a contribution originating from changes to phonon modes (shown as shaded areas over the Drude spectrum). The combination of both contributions can successfully reproduce the experimental data (dots). We, therefore, conclude that the deviations from the Drude response in the OPTP spectra of phase-segregated perovskite originate from higher frequencies of the halide sub-lattice vibrations in the excited state compared to the ground state.

**Fig. 2:**
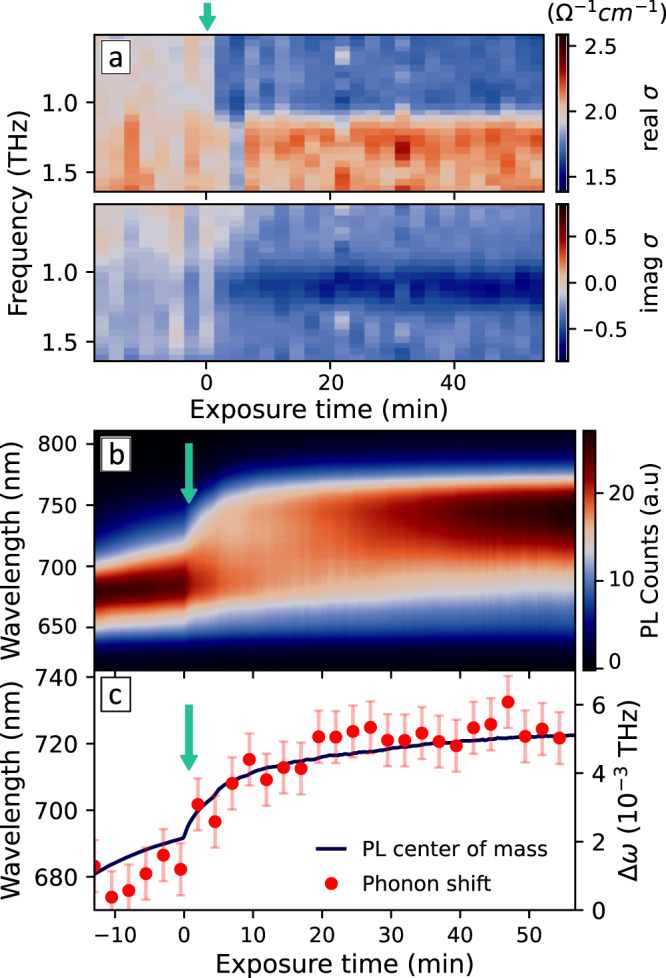
Simultaneous observation of halide segregation and enhanced phonon anharmonicity. **a** Evolution of the THz photoconductivity spectra taken at 10 ps delay after 400 nm photoexcitation. The green arrow indicates when exposure to the 532 nm CW laser starts (at ${t}_{{{{{{{{\rm{exposure}}}}}}}}}=0$), inducing phase segregation. The intensity of the CW laser was slowly ramped from 0 to 500 mW/cm^2^ over the experiment to obtain a gradual segregation process. **b** PL spectral evolution of the MAPb(I_0.5_Br_0.5_)_3_ thin film during segregation. **c** PL centroid (i.e., weighted mean, blue) and photoexcitation-induced phonon frequency shift Δ*ω* (red dots) extracted from fits to the THz photoconductivity spectra (at *t*_pump_ = 10 ps) during phase segregation. Error bars represent the average standard deviation of the fit.

Lattice anharmonicity is an intrinsic property of metal halide perovskites^32,33^ and is therefore also present in the neat triiodide material (see Supplementary Figs. 16 and 17). However, interestingly, these phonon anharmonicity features are substantially enhanced in the mixed halide film after phase segregation (Fig. 1f, as opposed to the plain Drude response in Fig. 1e), presenting us with a powerful tool for directly monitoring halide segregation. To visualise this phenomenon we recorded the frequency-resolved photoconductivity spectra during the phase segregation process along the simultaneously acquired steady-state PL of a MAPb(I_0.5_Br_0.5_)_3_ film. Figure 2 shows the evolution of Δ*ω* extracted from the analysis of the most highly resolved 1-THz mode in the OPTP spectra (see analysis of concomitant changes to *ω*_0_ in Supplementary Note 1). Phase segregation is induced with a 532 nm CW laser starting at ${t}_{{{{{{{{\rm{exposure}}}}}}}}}=0$ (indicated by a green arrow), during which we clearly observe an enhancement of Δ*ω* that follows the shift observed in PL spectra (Fig. 2c).

One can expect the phase segregation to affect the vibrational properties of the lattice. However, the dark phonon spectrum (where photoexcitation-induced anharmonicity is not present) shows no significant change after segregation (Supplementary Fig. 2). This finding is in agreement with the reports of segregation occurring in only a small portion of the material, leaving the majority of the mixed halide perovskite volume unaffected^2,9,17^. However, despite the limited scale of structural transformations, the PL spectrum undergoes a dramatic change, as most of the emission originates from the narrow-bandgap I-rich domains into which the charge carriers can funnel^7,39^. As a result of such funnelling, a substantial contribution to the total photoconductivity originates from charge carriers located in the narrow-bandgap domains. The modulations to the OPTP spectra upon phase segregation result from a differential measurement in transmission and are able to reveal such relatively small frequency shifts superimposed with the free charge-carrier conductivity response. The enhancement of Δ*ω* apparent in the photoconductivity spectra thus directly reflects the change in lattice properties experienced by charge carriers after they have funnelled into the I-rich phase. We can therefore associate the enhancement of Δ*ω* upon phase segregation shown in Fig. 2 with the presence of a substantial charge-carrier concentration in the I-rich domains. This association is further supported by a saturation effect with increasing photoexcitation density, which is analogous to the saturation observed in the PL spectra (see Supplementary Fig. 9 and Supplementary Note 3). Having established that the phonon shifts result from the presence of charge carriers in the I-rich phase, we further show below how such features reflect charge funnelling, enabling us to directly record the timescales over which such process occurs.

**Fig. 3:**
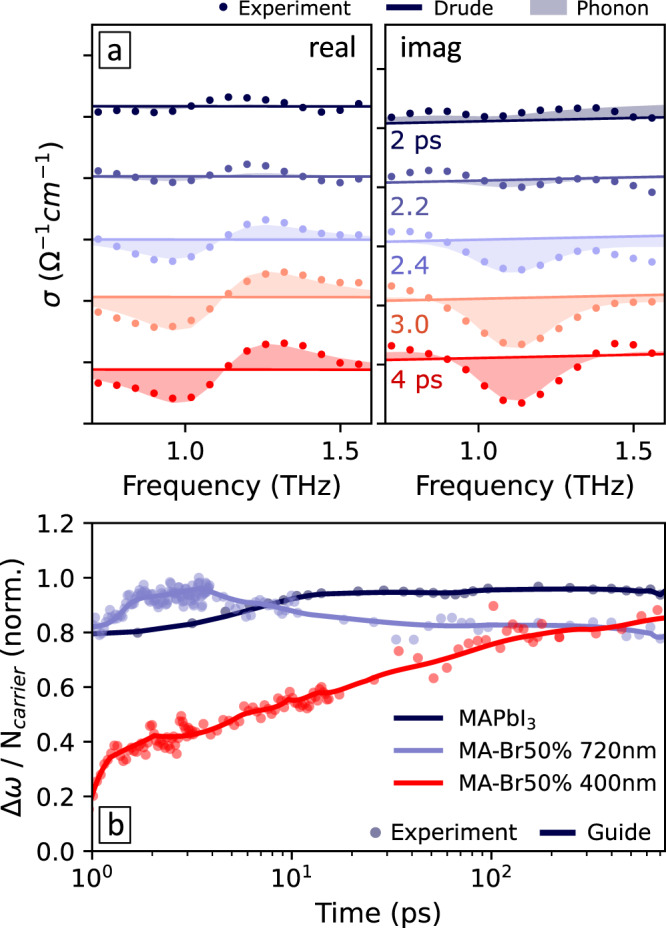
Picosecond evolution of phonon anharmonicity features in OPTP photoconductivity following pulsed photoexcitation. **a** THz photoconductivity spectra at various time delays following 400 nm photoexcitation of the MAPb(I_0.5_Br_0.5_)_3_ thin film after phase segregation. The spectra were offset for clarity. **b** Photoexcitation-induced phonon frequency shift Δ*ω* divided by carrier density *N* (and subsequently normalised for comparison), extracted from fits to the photoconductivity spectra as a function of time delay following photoexcitation. Dots show the fitted values extracted from data of a MAPbI_3_ thin film with 400 nm photoexcitation (dark blue), MAPb(I_0.5_Br_0.5_)_3_ thin film with 400 nm (red), and 720 nm pump (light blue), respectively. Solid lines are splines to guide the eye.

Figure 3a show examples of photoconductivity spectra recorded as a function of pump delay time *t*_pump_ following 400 nm photoexcitation over the fs-ps timescale and their respective fits to the phonon shift model (as described in Supplementary Note 1). Figure 3b displays the recorded dynamics in the fitted values of Δ*ω* normalised by charge-carrier densities *N* (extracted from the amplitude of the Drude response) to account for the population decay by recombination. We are disregarding early-time (<2ps) variations of phonon contributions previously reported to be associated with scattering mechanisms^38^. We find a gradual rise in Δ*ω*/*N* with increasing *t*_pump_ following 400-nm photoexcitation, consistent with the accumulation of charges within the I-rich phase on the timescale of tens of picoseconds, reflecting the presence of halide-segregated domains throughout the film’s nanoscale^7,39^. In contrast, the photoconductivity spectra of a MAPbI_3_ film and the phase-segregated mixed halide film with direct photoexcitation of the I-rich phase (also plotted for reference in Fig. 3b) show almost no variation of Δ*ω*/*N* over time delay *t*_pump_.

Overall, the combined dynamic shifts illustrated in Figs. 2 and 3 confirm that the concentration of charge carriers within phase-segregated I-rich domains is associated with enhanced phonon anharmonicity. This observation can be understood as either a cause or a consequence of halide segregation. On the one hand, lattice anharmonicity can be a cause of segregation, given that such lattice distortions in the photoexcited state facilitate ionic displacements. The funnelling of charge carriers to regions where phonon anharmonicity is stronger agrees with the preferential formation of segregated domains at surfaces, grain boundaries, or defective regions that experience a softening of the lattice potential and are more likely to allow halide segregation^25,40^. On the other hand, the formation of boundaries and lattice distortions can be a consequence of halide demixing. Bischak et al. suggested that large polarons forming randomly in the perovskite lattice may nucleate I-rich clusters, which then migrate to boundaries to relieve strain^8^. Within this picture, the enhanced phonon shifts we observe may derive from the formation of I-rich clusters that may stabilise large polarons. In addition, given that the anharmonicity effects are intensified by higher charge-carrier densities, the concentration of charge carriers within the I-rich phase can play a significant role in the observed phonon shifts (see Supplementary Note 3). These interpretations thus have an interesting implication for the structural stability of mixed halide perovskites, because such an enhanced anharmonicity may further promote ionic displacement to support halide segregation and other illumination-induced transformations^33,41–44^. Further investigations regarding the relationships between lattice anharmonicity and the material morphology, crystallinity,^45^, and composition (Supplementary Fig. 6), will provide valuable guidance for the development of more stable mixed halide perovskites, with enhanced stability against phase segregation.

### Recombination dynamics

**Fig. 4:**
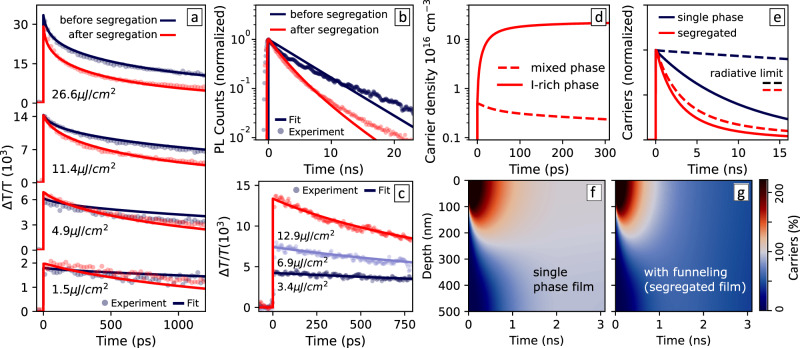
Impact of phase segregation on the charge-carrier dynamics. **a** OPTP transients of an MAPb(I_0.5_Br_0.5_)_3_ thin film at various photoexcitation fluences, before and after phase segregation. **b** PL dynamics of 670 nm emission before segregation (dark blue) and 750 nm after segregation (red), at fluence 50 nJ/cm^2^. **c** OPTP transients of an MAPb(I_0.5_Br_0.5_)_3_ thin film after segregation, with direct photoexcitation of the I-rich domains at 720 nm. In **a**–**c**, dots are experimental data and solid lines are fits to the model described in Supplementary Note 2. **d** Charge-carrier densities within each phase of the segregated film, extracted from the model described in Supplementary Note 2. **e** Simulated decay in a single-phase film before segregation (dark blue) and after segregation (red). Dashed lines are dynamics simulated with no trap-assisted recombination (*k*_1_ = 0). **f**, **g** Simulated charge-carrier distribution over time across a 500 nm thick film at the radiative limit (*k*_1_ = 0) before and after phase segregation. The initial distribution is given by the absorption profile of 400 nm light. The carrier population is normalised by the average initial density so that 100% value is equivalent to the uniform distribution of the photogenerated carriers following diffusion. Values below 100% after reaching a uniform distribution are a result of carrier loss by radiative recombination.

Following the observation of fast charge funnelling we are able to analyse the impact of phase segregation on the subsequent charge-carrier recombination dynamics in the mixed halide perovskite. We first examined the PL decays obtained with 400-nm photoexcitation at the low fluence of 50 nJ/cm^2^ (Fig. 4b). The PL lifetime was found to be shorter in the phase-segregated film, with *τ*_PL_ = 2 ns (acquired at 750 nm) in contrast with *τ*_PL_ = 4 ns (at 670 nm) before segregation. Spectrally resolved PL dynamics was unable to reveal the presence of high energy emission or the early-time rise of the low-energy peak (Supplementary Fig. 11), in agreement with our previous observation of fast charge funnelling into the I-rich domains (Fig. 3), which occurs mainly within the experiment resolution of ~1 ns.

To evaluate the recombination dynamics at higher charge-carrier densities and higher time resolution, we acquired frequency-averaged OPTP transients as a function of pump time delay *t*_pump_ before and after phase segregation (Fig. 4a). While the amplitudes of Δ*T*/*T* at *t*_pump_ = 0 (associated with the initial charge-carrier mobilities) are not affected, the transients show faster decays after halide segregation. To better evaluate this change, the OPTP transients were fitted according to

1 $$\frac{dn}{dt}=-{k}_{1}n-{k}_{2}{n}^{2}-{k}_{3}{n}^{3},$$

which describes the decay of charge-carrier density *n* following pulsed photoexcitation as a combination of first-order recombination (given by rate constant *k*_1_), second-order radiative recombination (given by *k*_2_), and third-order processes (such as Auger, given by *k*_3_)^11–13^. To improve the accuracy of the model, charge-carrier diffusion and photon recycling effects have also been implemented^46^ (see Supplementary Note 2 for details). Because of the limited time range of the OPTP experiment, *k*_1_ was extracted from the PL dynamics (Fig. 4b). The shorter lifetimes in the phase-segregated material translate as higher values of *k*_1_. However, this enhancement of the first-order recombination rate does not reproduce the faster decays of the OPTP transients observed at higher excitation intensities. Instead, we also observe an enhancement in the second-order decay rate constant *k*_2_. We highlight that our recent studies carried out on identical films show no formation of PbI_2_ or changes to the crystallinity of the material during phase segregation, which rules out any influence of degradation processes^17^. Furthermore, while variations in the trap density or charge funnelling dynamics can affect *k*_1_, this enhancement of *k*_2_ is rather unexpected, since band-to-band bimolecular recombination should be an intrinsic property of a semiconductor^12,46^. I-rich perovskites such as those forming domains where most of the radiative recombination occurs are in fact characterised by lower intrinsic values of *k*_2_ than their bromide counterparts^6,47^.

To understand the enhancement of *k*_2_ upon phase segregation, we first consider photon reabsorption effects, which have been demonstrated to significantly impact the perceived bimolecular recombination rates^46,48^. The suppressed photon recycling associated with the red-shifted emission after phase segregation can result in a higher effective value of *k*_2_. However, this effect has been accounted for in our analysis, demonstrating that the enhanced recombination rates do not originate from reabsorption effects.

We then consider the confinement of charge carriers in small particles/domains as a possible cause of the enhanced higher-order recombination rates^24^. To investigate the recombination dynamics within the segregated domains we performed the OPTP experiment with direct photoexcitation into the I-rich phase. Figure 4c shows the OPTP transients after 720 nm photoexcitation, where the solid lines are fits according to Eq. (1). The value of *k*_2_ extracted for direct photoexcitation of I-rich domains is comparable to that for the mixed-phase film before segregation (see Table 1), suggesting that confinement in I-rich domains is not the cause of the increased value of *k*_2_ that is obtained with 400 nm photoexcitation into the phase-segregated film. This is in agreement with the high mobilities and Drude-like response of the THz photoconductivity in the I-rich phase (Fig. 1g), which indicate that charge carriers are not strongly confined within these domains. From these combined observations we conclude that quantum confinement-related enhanced recombination cannot be responsible for the apparent increased bimolecular recombination in the phase-segregated film.

**Table 1:** Recombination rate constants.

Material	*μ* (cm^2^ V^−1^ s^−1^)	*k*_1_ (10^6^ s^−1^)	*k*_2_ (10^−9^ cm^3^ s^−1^)
Mixed phase (before segregation)	37.3 ± 2.7	88 ± 2	2.7 ± 0.1
Majority phase	37.2 ± 0.6	88*	2.7*
I-rich phase	49 (35 < *μ* < 66)	88*	2.5 (1.9 < *k*_2_ < 6)
Phase-segregated (effective values)	37.2 ± 0.6	170 ± 10	4.6 ± 0.2

Values marked by * have not been directly obtained from experimental data but have been adopted for modeling the recombination dynamics. For simplicity, *k*_3_ was fixed to 1 × 10^−28^ cm^6^ s^−1^ for all fits and simulations. The lower and upper boundaries (accounting for uncertainties in the calculation of charge-carrier dynamics) for *μ* and *k*_2_ in the I-rich phase have been detailed in Supplementary Note 6. Intervals of confidence are the standard deviation of at least ten measurements of *μ*, and the standard deviation of the recombination rates obtained from fits to the charge-carrier dynamics.

Finally, we consider the impact of charge funnelling and increased charge-carrier density within the I-rich domains on the recombination dynamics. For this purpose, we developed an adapted model that assumes the semiconductor film to comprise a majority mixed-halide phase interspersed with an I-rich phase occupying *R*_vol_ = 1% of the total volume of the film (based on previous studies^2,9^). Photogenerated charge carriers can recombine within each phase or be transferred from the majority phase into the I-rich phase (a back-transfer rate was implemented to account for saturation effects). Although extended illumination can affect the trap-assisted recombination rates in metal halide perovskites^42,49^, these effects are minimised by film encapsulation and low CW intensities in our experiments (see Supplementary Note 4). We, therefore, fixed the first-order recombination rate constant *k*_1_ to the values obtained before phase segregation. Table 1 summarizss the values obtained from fits to experimental data and the values adopted for modeling the dynamics (see Supplementary Note 2 for full details of the model).

Figure 4a shows the fits to the adapted charge funnelling model (red solid lines) with good agreement to experimental data for the phase-segregated film (red dots). Our model also well reproduces the PL dynamics (Fig. 4b), demonstrating how charge-carrier funnelling can explain the shortening of their lifetimes in the phase-segregated film with no change to any of the intrinsic recombination rate constants required, including the rates associated with trap-assisted recombination. The excellent fit between this model and the experimental data demonstrates that shorter lifetimes are rather a result of the increased charge-carrier concentration within the segregated I-rich domains, as shown in Fig. 4d. The electron-hole bimolecular recombination (given by *k*_2_*n*^2^) is enhanced by this increase in charge-carrier density (*n*), leading to the observation of higher *effective* values of *k*_2_.

While our charge funnelling model does not account for the presence of intermediate halide compositions and heterogeneous distribution of I-rich domains through the phase-segregated material, it reproduces with excellent agreement a series of phenomena. In addition to the OPTP transients (Fig. 4a) and the changes in PL lifetimes (Fig. 4b), our model also correctly reproduces the saturation effects observed in the PL spectra with increasing illumination intensities (Supplementary Figs. 10 and 14), and is consistent with the dynamic changes of the THz photoconductivity spectral features (Supplementary Fig. 15). Therefore, our analysis successfully demonstrates how the presence of phase-segregated domains in mixed halide perovskites dominates the charge-carrier dynamics in these materials even upon minimal transformations to the film structure and to intrinsic optoelectronic properties, such as charge-carrier mobilities and recombination rate constants.

### Implications of charge funnelling to device performance

One important figure of merit often highlighted with regards to photovoltaic device performance is the requirement for high PL quantum efficiency (PLQE), given that a high PLQE reflects reduced trap-mediated recombination losses. However, as we show below, for halide-segregated materials, an enhanced PLQE may instead simply derive from increased bimolecular recombination associated with charge funnelling. Such radiative efficiency enhancements upon halide segregation are therefore not necessarily advantageous to photovoltaic device operation. To unravel such effects, we use the parameters obtained from the OPTP analysis to simulate the excited-state dynamics at low initial photoexcitation density (~10^16^ cm^−3^), similar to solar cell operation conditions at one-sun illumination, for a mixed halide film before and after phase segregation. Our model allows us to extract the total number of photons emitted by both phases and the resulting PLQE values. We obtain predicted PLQE = 4% for the mixed-halide film before segregation, and 65% for the segregated film, in fair agreement with the commonly observed enhancement of radiative efficiency of mixed halide perovskites following phase segregation^9,14,50^. However, while high PLQE is often seen as positive for photovoltaic device performance, this reciprocity relation is not valid in phase-segregated mixed halide perovskites^10^. Efficient extraction of charges in a solar cell also requires sufficiently long charge-carrier lifetimes and diffusion lengths. To better assess the impact of charge funnelling we compare in Fig. 4e the simulated decay of charge-carrier population in the single-phase film before segregation and in the phase-segregated film where funnelling occurs. The dashed lines are the simulated dynamics at the radiative limit where *k*_1_ = 0, i.e., in the absence of trap-assisted recombination losses. It is clear that even at the radiative limit the charge-carrier population in the phase-segregated film (red) decays dramatically faster with respect to the non-segregated film (dark blue). Such drastic shortening of lifetimes indicates that radiative recombination losses are likely to suppress charge collection and hence the efficiency of mixed-halide solar cells after phase segregation, in addition to any losses associated with trap-assisted recombination.

Overall, while we have demonstrated that the charge-carrier mobilities of mixed-halide perovskites are not impacted by phase segregation, the charge-carrier diffusion lengths can be substantially reduced because they scale according to the square root of the recombination lifetimes. We highlight such an effect by showing in Fig. 4f, g the simulated charge-carrier distribution across a 500-nm thick perovskite film as a function of time delay following 400-nm photoexcitation. In a single-phase (unsegregated) film, the photogenerated carriers can reach a uniform distribution in ~3 ns while retaining 97% of the total initial population (Fig. 4f). In contrast, in the phase-segregated film, charge accumulation in I-rich domains detrimentally limits diffusion because over 50% of initial charge carriers are lost to recombination by 3 ns after photogeneration (Fig. 4g). Our analysis further shows that such losses are aggravated by the red shift in the emission of the I-rich domains, which results in low efficiency of photon recycling within the mixed-halide perovskite layer. While the overlap between the absorption and emission spectra of the MAPb(I_0.5_Br_0.5_)_3_ film before segregation results in a predicted ratio of photon recycling above 40%, we estimate a recapture efficiency below 20% after segregation. For single-junction solar cells made of mixed-halide perovskites, the enhanced radiative recombination and shortening of diffusion lengths in phase-segregated material will be detrimental to photovoltaic performance particularly in thicker films, where the extraction of charge carriers fails to compete effectively with the fast recombination process introduced by charge-carrier funnelling into I-rich domains^51^.

We note that our analysis unveils several opportunities to limit the detrimental effects of halide segregation through the targeted design of photovoltaic device architectures. A focus on reducing charge extraction losses through careful optimisation of the mixed-halide absorber thickness may ensure that charge collection is not outperformed by the enhanced recombination. Furthermore, photon management may potentially alleviate a significant fraction of the additional recombination losses introduced by halide segregation, given that these result from a radiative electron-hole recombination process. While we have shown that low-energy photons originating from the I-rich phase are not normally captured efficiently by the predominantly wide-bandgap material, reabsorption could be boosted by limiting light outcoupling through interface engineering. Although enhancing photon recapture would not have a great impact in mitigating V_OC_ losses, we highlight that such losses have been shown to arise mostly from trap-assisted recombination rather than bandgap segregation^9^. Therefore, efforts to improve material processing and reduce the density of defects in combination with light management strategies have great potential to promote the viability of mixed-halide perovskite-based solar cells in spite of halide segregation. Even more promising opportunities for photon recycling are presented when mixed-halide perovskites are incorporated into a multi-junction architecture, which is likely to be the prime applications target for these wide-band-gaps semiconductors. As part of an overall light managing strategy within a complete multi-junction solar cell, low-energy photons emitted by the I-rich phase of the mixed-halide perovskite layer may be effectively recaptured within the narrow-bandgap cell of the tandem device. Such a transfer process between different absorber layers in the device could occur through either photon emission and reabsorption^46^, or direct resonance energy transfer^52^. In this scenario, the combined multi-junction performance could compensate for some of the losses incurred in the wide-bandgap mixed halide absorber, provided the overall architecture is optimised taking such transfers into account.

In conclusion, we have assessed how phase segregation in mixed halide perovskites affects their optoelectronic properties, and may in turn impact the performance of photovoltaic devices. We report that, surprisingly, halide segregation causes negligible changes to the THz effective charge-carrier mobilities of MAPb(I_0.5_Br_0.5_)_3_ perovskite. This observation is confirmed through direct photoexcitation of the I-rich domains and finding the resulting charge-carrier mobility values to be very similar, or even higher than those obtained for the majority (unsegregated) phase. Accordingly, THz photoconductivity spectra are Drude-like, revealing an absence of strong charge-carrier localisation within the narrow-bandgap I-rich domains. However, the THz spectra further reveal the presence of phonon features associated with lattice anharmonicity, which are enhanced upon halide segregation. Such phonon-related features in the photoconductivity spectra reflect the charge funnelling and increased concentration of charge carriers within I-rich domains, and highlight how ionic displacement can be promoted by lattice anharmonicity in these heterogeneous materials. Through analysis of OPTP transients, we demonstrate that charge funnelling into I-rich domains leads to locally enhanced charge-carrier concentrations that cause an increase in effective radiative recombination rates and luminescence intensities. Our analysis demonstrates that the small structural transformations occurring during phase segregation have a dramatic effect on the charge-carrier dynamics in mixed halide perovskites, even when fundamental properties such as charge-carrier mobilities and recombination rate constants remain unchanged. Our findings show that charge funnelling into segregated domains may cause enhanced “radiative losses” and lower charge-carrier diffusion lengths, which, in the absence of appropriate mitigation strategies, would lower the performance of photovoltaic devices. We, therefore, propose several approaches with which such effects may be partly compensated, including careful optimisation of the absorber thickness to maintain charge extraction, and light management strategies that optimise the recapture of photons emitted through the accelerated radiative recombination of charge carriers after funnelling into I-rich regions of the phase-segregated mixed-halide perovskite film. Combined with strategies targeted to reduce halide segregation^3^, such approaches may pave the way for the implementation of mixed-halide perovskites films in efficient multi-junction solar cells.

## Supplementary information

Supplementary Information

## Data Availability

The datasets generated during and/or analysed during the current study are available from the corresponding author on reasonable request.
