# Supplementary material for: Phase segregation in mixed-halide perovskites affects charge-carrier dynamics while preserving mobility
Source: Nat Commun. 2021 Nov 29;12:6955. doi: 10.1038/s41467-021-26930-4 (PMC8630172; doi:10.1038/s41467-021-26930-4)
Supplement: Supplementary file 1 — Supplementary Information [file 41467_2021_26930_MOESM1_ESM.pdf]

Phase segregation in mixed-halide perovskites affects charge-carrier dynamics while preserving mobility

Silvia G. Motti,[1] Jay B. Patel,[1] Robert D. J. Oliver,[1] Henry J. Snaith,[1] Michael B. Johnston,[1] Laura M. Herz,[2,1*]

[1] Department of Physics, University of Oxford, Clarendon Laboratory, Parks Road, Oxford OX1 3PU, United Kingdom

[2] TUM Institute for Advanced Study, Technische Universität München, Lichtenbergstr. 2a, 85748 Garching bei München, Germany

* Corresponding author: laura.herz@physics.ox.ac.uk

Supplementary methods

Sample Preparation

Substrate cleaning: z-cut quartz substrates were washed using Hellmanex, acetone, isopropanol and ethanol. The quartz substrates were then placed in the oxygen plasma asher for 10 minutes.

Perovskite film fabrication: $CH_3NH_3Pb(I_{0.5}Br_{0.5})_3$ perovskite thin films were prepared using the acetonitrile route as reported previously in Knight et al, Advanced Energy Materials 2020, 10, 1903488. Briefly, 0.5 M CH_3NH_3I (GreatCell), 0.5 M CH_3NH_3Br (GreatCell), 0.5 M PbI_2 (TCI) and 0.5 M $PbBr_2$ (Alfa Aesar) were weighed out and dissolved in acetonitrile (Sigma Aldrich) as described by Noel et al.[1] The solution was then spun onto the z-cut quartz in a nitrogen filled glovebox at 2000 rpm and the films were then annealed on a hot plate inside a glovebox for 1 hour at 100 °C .

PMMA encapsulation: The perovskite films were then coated with a layer of PMMA (poly(methyl methacrylate) to reduce the impact of atmospheric effects.[2] PMMA was purchased from Sigma Aldrich, mean molar weight 97000. PMMA was dissolved in chlorobenzene at a concentration of 150 mg/mL. The PMMA film was formed by dynamically depositing 40 μL of the solution on to the perovskite film at 2000 rpm. The substrate continued to spin for 25 s following this deposition. The films were then annealed at 100 °C for 1 min to drive off any residual solvent.

A parallel study by Knight et al. has been carried out in parallel to this work, using identical $CH_3NH_3Pb(I_{0.5}Br_{0.5})_3$ samples prepared in the same batch as the ones used here, and presents a detailed analysis of the XRD diffraction during phase segregation.[3]

Steady-State Absorption

Reflection and transmission spectra were measured using a Bruker 80v Fourier-transform infrared spectrometer with a tungsten halogen lamp source, a CaF_2 beamsplitter and a silicon diode detector. Data was collected with a resolution of 4 cm^{-1}. A silver mirror was used as reflection reference and a blank quartz substrate was used as transmission reference.

Steady-State and Time-Resolved Photoluminescence

The excitation source was a 398 nm pulsed diode laser (PicoHarp, LDH-D-C-405M) operating at 10 MHz. The PL was collected in reflection geometry and coupled into a grating spectrometer (Princeton Instruments, SP-2558). Time-resolved measurements were acquired in a Time Correlated Single Photon Counting (TCSPC) system (PicoHarp300). The measurements were performed under vacuum (pressure $<10^{-1}$ mbar).

Optical-Pump Terahertz-Probe (OPTP) Spectroscopy

Optical-Pump THz-Probe (OPTP) measurements were performed using a Spectra Physics Mai Tai-Empower-Spitfire Pro Ti:Sapphire regenerative amplifier. The amplifier generates 35 fs pulses centered at 800 nm at a repetition rate of 5 kHz. The optical pump excitation was obtained by frequency doubling the fundamental laser output through a BBO crystal, resulting in 400 nm pulses. THz probe pulses were generated by a spintronic emitter which was composed of 1.8 nm of $Co_{40}Fe_{40}B_{20}$ sandwiched between 2 nm of Tungsten and 2 nm of Platinum, all supported by a quartz substrate. Detection of the THz pulses was performed using electro-optic sampling in a (110)-ZnTe crystal (thickness 1 mm). The FWHMs of the beams for the pump and THz pulses at the sample were measured to be 2 mm and 0.5 mm respectively. The phase segregation of the mixed halide samples were induced using a 532 nm continuous wave laser. The CW laser was coupled into a fiber and directed onto the sample over a spot size sufficiently large (FWHM of 1.6 mm) to ensure coverage of the area probed by the THz pulse. The sample, THz emitter and THz detector were held under vacuum ($<10^{-2}$ mbar) during the measurements.

The effective charge-carrier mobility was extracted from the OPTP data using the method previously described by Wehrenfennig et al.[4] In brief, the sheet photo-conductivity, ΔS, of a material with a thickness much shorter than the wavelength of the THz radiation can be expressed as

$$\Delta S = -\varepsilon_0 c(n_a + n_b)(\frac{\Delta T}{T}) \tag{1}$$

where c is the speed of light, ε_0 is the vacuum permittivity, and n_a and n_b are the THz refractive indices of

the materials interfacing the perovskite layer at the front and rear respectively. The quantity $\Delta T/T$ is the ratio of the photo-induced change in THz electric field to the transmitted THz electric field in the dark. The initial number of photo-excited charge carriers N is given by

$$N = \phi \frac{E\lambda}{hc}(1 - R_{pump} - T_{pump}) \tag{2}$$

with E being incident pump pulse energy, λ the excitation wavelength, ϕ the ratio of free charges created per photon absorbed, and R_{pump} and T_{pump} being the reflected and transmitted fractions of the pump beam. These two equations can be used to extract the charge-carrier mobility μ through

$$\mu = \frac{\Delta S A_{eff}}{Ne} \tag{3}$$

where A_{eff} is the effective area from the overlap of the pump and probe beams and e is the elementary charge. Substituting Equations 1 and 2 into Equation 3 we obtain

$$\phi\mu = -\frac{\varepsilon_0 c(n_a + n_b)(A_{eff})}{Ne\lambda(1 - R_{pump} - T_{pump})}\left(\frac{\Delta T}{T}\right) \tag{4}$$

from which the effective charge-carrier mobility $\phi\mu$ may be determined based on the pump beam parameters and the initial measured $\Delta T/T$ of the sample. Here, μ is the charge-carrier mobility, and ϕ is the charge-to-photon branching ratio. In this work we assume ϕ to be equal to one, i.e., we neglect the presence of excitons at room temperature, in accordance to the low exciton binding energies in lead halide perovskite semiconductors.[5,6] Charge-carrier mobility values were calculated based on the average of at least 10 measurements.

Supplementary Note 1. THz spectra

The effective photoconductivity $\Delta\sigma(\omega)$ is normally calculated from the pump-induced changes to the THz transmission by:

$$\sigma_{photo}(\omega) = -\frac{\varepsilon_0 c(n_1 + n_2)}{L}\frac{\Delta T}{T} \tag{5}$$

where L is the semiconductor film thickness. This formula is valid when the THz transmission of the semiconductor in the dark is approximately equal to the transmission of the substrate. However, this approximation is not valid for THz frequencies near phonon resonances, as has been demonstrated by La-o-vorakiat et al.,[7] or for highly doped semiconductor films, as demonstrated by Ulatowski et al.[8]

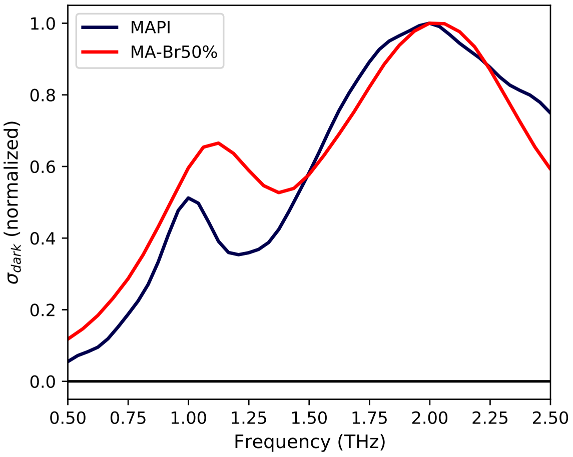

Supplementary Figure 1 Phonon contribution to the THz spectra of $MAPbI_3$ and $MAPb(I_{0.5}Br_{0.5})_3$ in dark.

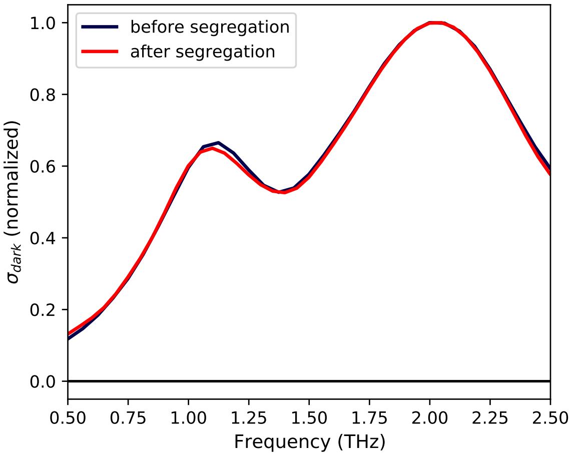

Supplementary Figure 2 Phonon contribution to the THz spectra in dark of $MAPb(I_{0.5}Br_{0.5})_3$ before and after segregation.

A more accurate approach for calculating σ from the experimental data takes into account the dark THz spectrum in the following equation:

$$\sigma_{photo}(\omega) = -\frac{T_{substrate}}{T}\frac{\varepsilon_0 c(n_1+n_2)}{L}\frac{\Delta T}{T+\Delta T} \quad (6)$$

where $T_{substrate}$ is the substrate transmission and T is the transmission of the sample (substrate and semiconductor) in dark. The approximation $\frac{\Delta T}{T+\Delta T} = \frac{\Delta T}{T}$ is still valid when $\Delta T << T$, such as in the cases presented in this study. However, the additional term $\frac{T_{substrate}}{T}$ results in significant corrections to the phonon features superimposed in the photoexcited spectra.

The dark THz spectra can be acquired using a blank quartz substrate as a reference. Figure 1 shows the dark spectrum of the $MAPb(I_{0.5}Br_{0.5})_3$ film in dark, where two phonon modes can be seen at $\sim$ 1 THz

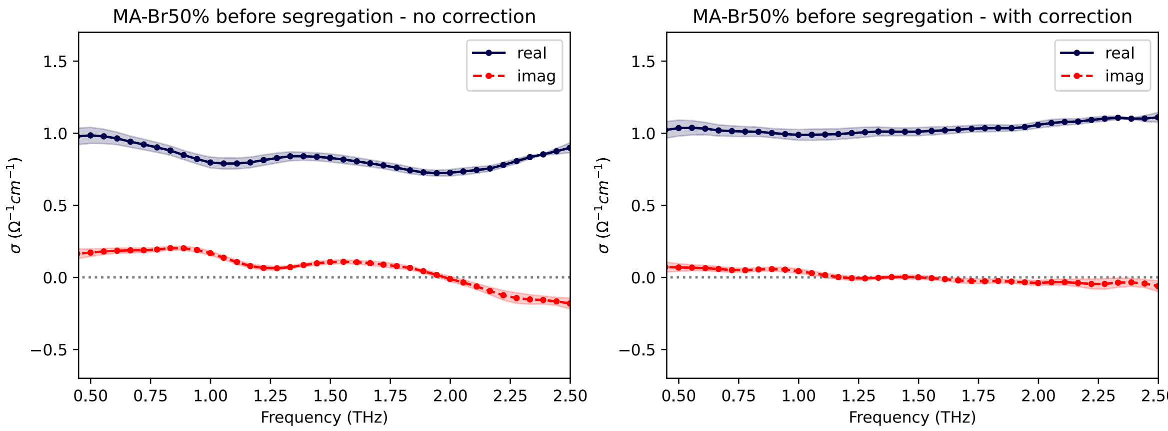

Supplementary Figure 3 THz photoconductivity spectra of the $MAPb(I_{0.5}Br_{0.5})_3$ film before phase segregation with 400 nm photoexcitation, with and without correction for the dark absorption of the phonon modes.

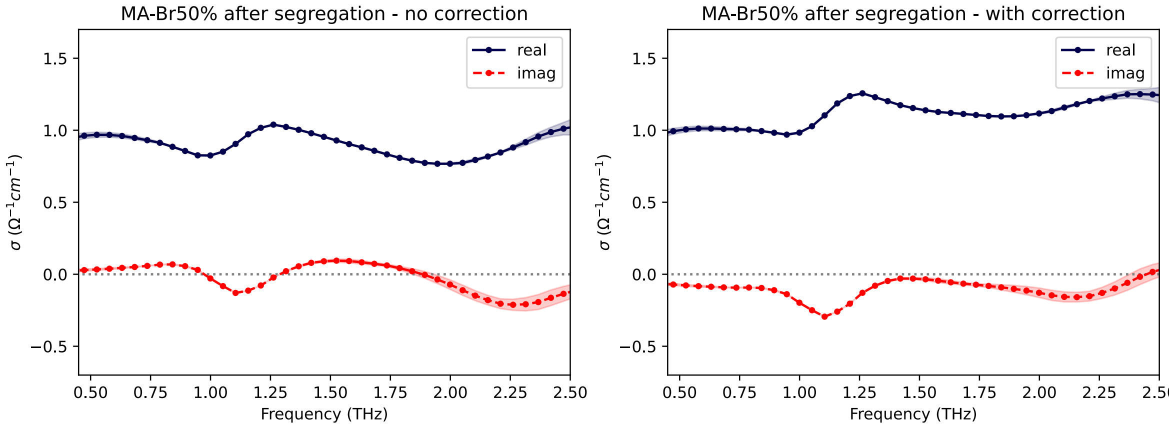

Supplementary Figure 4 THz photoconductivity spectra of the $MAPb(I_{0.5}Br_{0.5})_3$ film after phase segregation with 400 nm photoexcitation, with and without correction for the dark absorption of the phonon modes.

and 2 THz. These modes are slightly blue-shifted with respect to $MAPbI_3$. Upon phase segregation, no significant changes are observed in the spectra (Figure 2).

The correction factor $\frac{T_{substrate}}{T}$ obtained from the dark spectra was applied in the calculation of the photoconductivity. Figure 3 and 4 show $\sigma(\omega)$ with and without correction for the dark spectra of the $MAPb(I_{0.5}Br_{0.5})_3$ film before and after halide segregation. We can clearly observe that while the corrections remove some phonon features, they do not explain the appearance of additional features following halide segregation. We then assign the derivative shape of the real part of σ in Figure 4 to a shift in the phonon resonance frequency upon photoexcitation.

Modelling of THz photoconductivity

The photoconductivity σ can be described by the Drude model as

$$\sigma_{Drude}(\omega) = \frac{N_{carrier}e^2}{m^*}\frac{i}{\omega + i\gamma} \tag{7}$$

where m^* is the charge carrier effective mass and γ is the scattering rate, which relates to the mobility μ by

$$\mu = \frac{e}{m^*\gamma} \tag{8}$$

In the phase-segregated mixed halide films, the spectra deviate from the Drude response. The deviations can be described by a shift in the phonon modes, as described by Zhao et al.[9] The phonon frequency shift results in a derivative shape superimposed with the Drude response. The effect of the shift on the OPTP signal is illustrated in Figure 5.

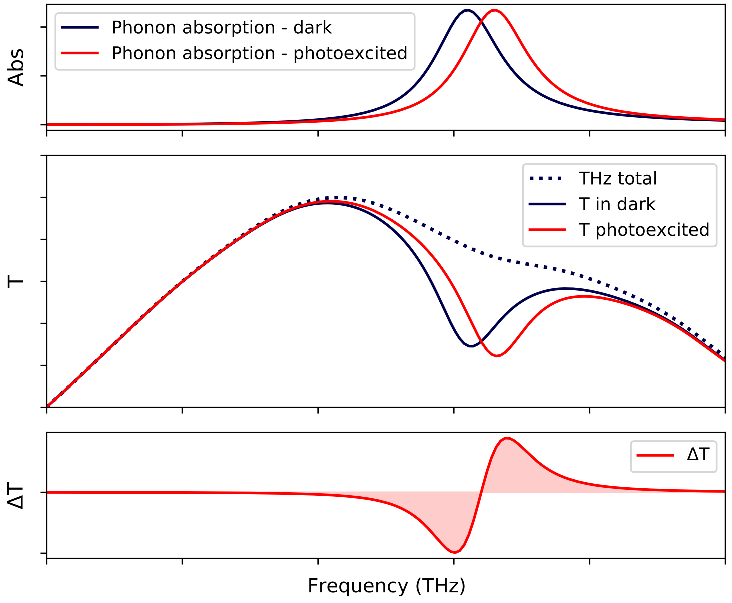

Supplementary Figure 5 Illustration of the effect of phonon shift observed in the photoconductivity spectra. The relative scale of the phonon shift has been intentionally exaggerated for illustration purposes.

The contribution of each phonon mode to the observed photoconductivity σ_{photo} is given by

$$\Delta\sigma_{phonon}(\omega) = \frac{\varepsilon_0\omega}{i(\omega_0^2 - \omega) + \omega\gamma}\Delta s + \frac{-2i\omega_0\varepsilon_0 S\omega}{(i(\omega_0 - \omega^2) + \omega\gamma)^2}\Delta\omega - i\varepsilon_0\omega\Delta\varepsilon_\infty \tag{9}$$

where $\Delta\omega$ is the photoinduced frequency shift, Δs is the change in mode weight, and $\Delta\varepsilon_\infty$ is the change in background dielectric constant. The centre frequency of the mode ω_0, the damping rate γ, and the mode amplitude (proportional to the oscillator strength) S are extracted from Lorentzian fits to the dark spectra

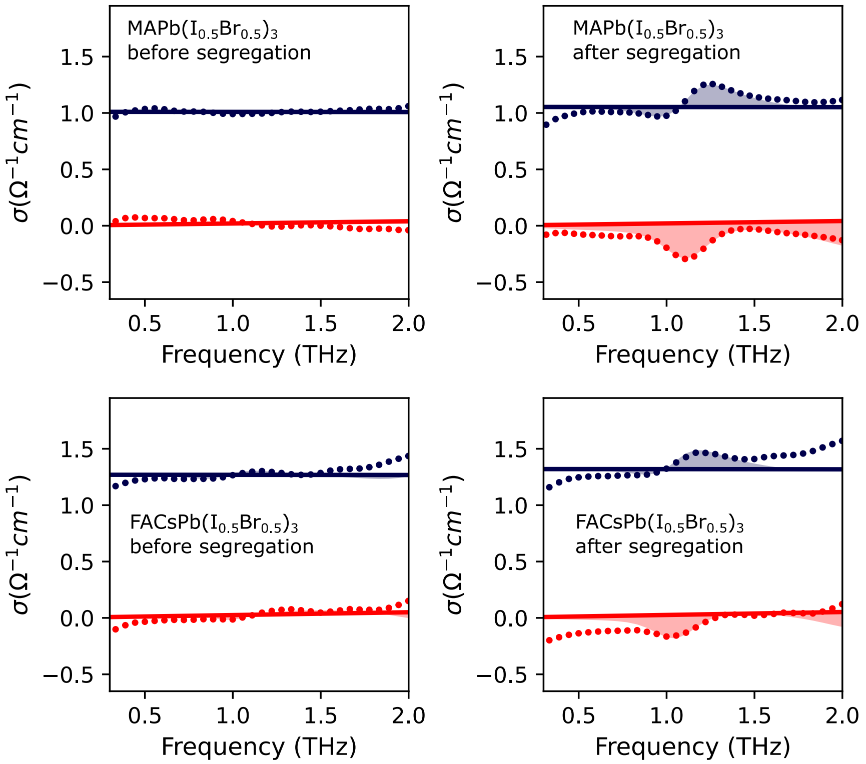

Supplementary Figure 6 THz photoconductivity spectra of $MAPb(I_{0.5}Br_{0.5})_3$ and $FACsPb(I_{0.5}Br_{0.5})_3$ films at 5 ps after photoexcitation with 400 nm pump before and after phase segregation. The Drude response is plotted as solid lines and the phonon contribution is shown as shaded areas over the Drude spectrum.

(Figure 1). The measured photoconductivity spectra is fitted to

$$\sigma = \sigma_{Drude} + \sigma_{phonon} \tag{10}$$

We have observed a small contribution of Δs compared to that of $\Delta\omega$ for reproducing the phonon features appearing in the photoconductivity spectra after phase segregation.

Impact of the organic cation

To confirm the reproducibility of the observations of phonon anharmonicity, we repeated the experiment with a $FACsPb(I_{0.5}Br_{0.5})_3$ film. Figure 6 shows the THz photoconductivity spectra with 400nm photoexcitation, before and after phase segregation, obtained for the FACs film, compared with the MA film (which was also presented in Figure 1 of the main text).

We can clearly observe that the phonon anharmonicity effects are enhanced after phase segregation in both materials. We also note that at similar photoexcitation intensities we obtain $\Delta\omega$=4.9×10^{-3} THz for the MA film after halide segregation and 3.6×10^{-3} THz for the FACs film. The less pronounced phonon

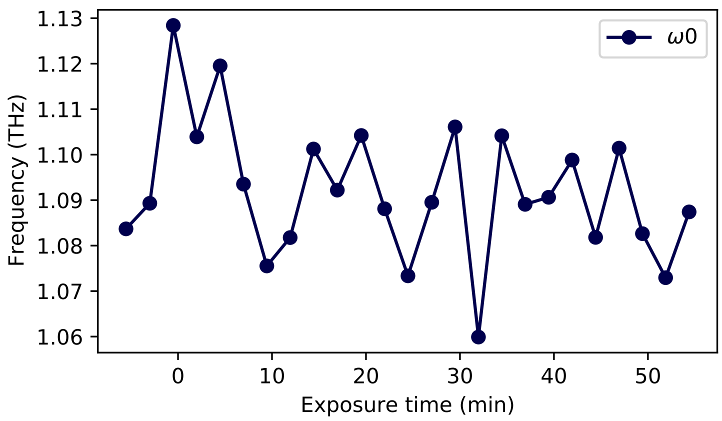

Supplementary Figure 7 Phonon resonance frequency ω_0 extracted from fits to the THz photoconductivity spectra acquired during phase segregation (shown in Figure 2 of the main text), as a function of CW light exposure time.

shift in the FACs film can be indicative of either higher stability of the material against halide segregation or lower susceptibility to lattice distortions in these mixed-cation compositions.[10]

Variations in ω_0

As the phonon frequencies depend on the mass of the vibrating atoms, an I-rich lattice has lower phonon frequencies compared to Br-rich compositions. With this relation in mind, we also allowed the equilibrium phonon resonance frequency ω_0 to vary during the fitting routine, to account for changes in the lattice composition experienced by charge-carriers during the halide segregation process (Figure 7) or during the funnelling dynamics (Figure 8). We observe a slight shift of ω_0 to lower frequencies during the fast funnelling (Figure 8), the changes to ω_0 extracted from the spectra at $t_{pump} = 5ps$ do not show significant variations above the uncertainty levels. This suggests that the phonon shifts originate mainly from regions with a constant lattice composition.

To evaluate the dependence of the anharmonicity effects on the charge-carrier densities we acquired the spectra at various photoexcitation intensities. Figure 9 shows the values of $\Delta\omega$ extracted from the fits to the spectra as a function of charge-carrier density $n_{carrier}$ (also extracted from the fits). While $\Delta\omega$ increases with increasing photoexcitation density, the ratio $\Delta\omega/N_{carrier}$ shows a saturation behaviour (consistent with the saturation of the low-bandgap emission observed in the fluence dependence of the PL spectra in Figure 10). The fits obtained for a $MAPbI_3$ film, on the other hand, result in lower $\Delta\omega$ and fairly constant $\Delta\omega/N_{carrier}$ with increasing photoexcitation density. This observation is consistent with the saturation of the I-rich phase in the phase-segregated $MAPb(I_{0.5}Br_{0.5})_3$ film (Figure 10) and the enhanced phonon anharmonicity effects in such I-rich domains.

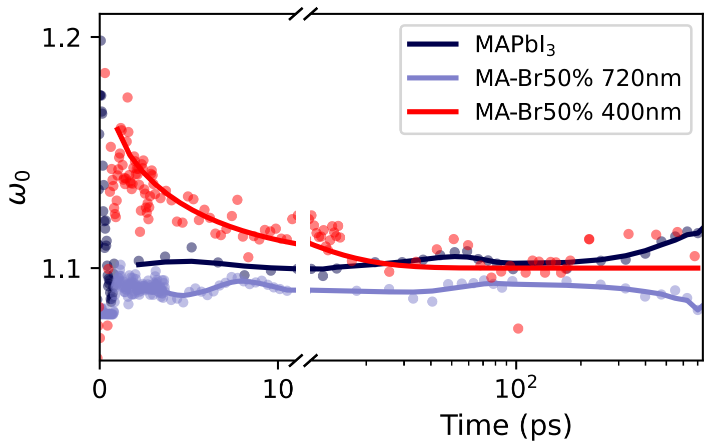

Supplementary Figure 8 Phonon resonance frequency ω_0 extracted from fits to the THz photoconductivity spectra acquired as a function of pump time delay t_{pump} following 400 nm photoexcitation into the phase-segregated $MAPb(I_{0.5}Br_{0.5})_3$ perovskite.

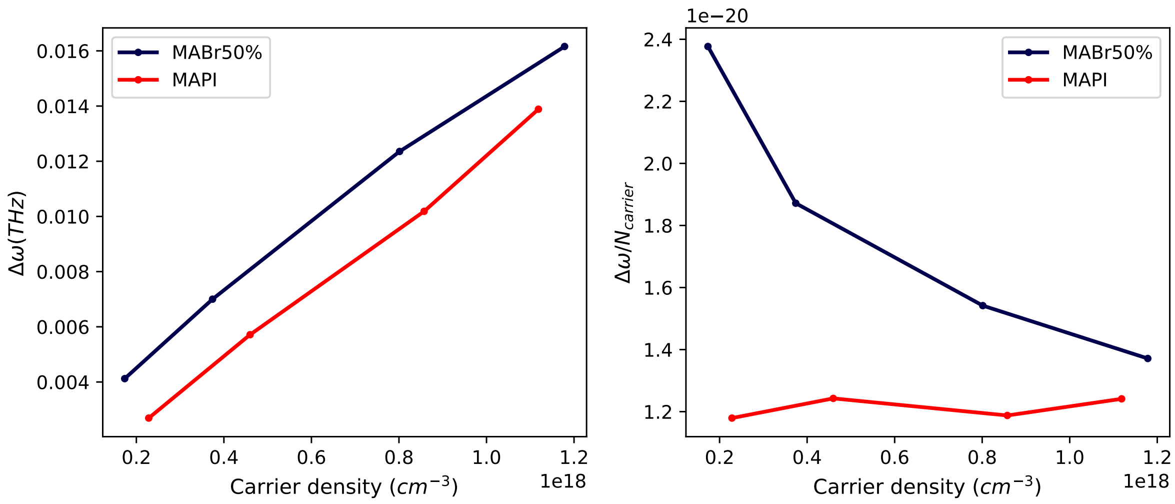

Supplementary Figure 9 Phonon shift $\Delta\omega$ extracted from the THz photoconductivity spectra of phase-segregated $MAPb(I_{0.5}Br_{0.5})_3$ and $MAPbI_3$ with 400 nm photoexcitation with increasing photoexcitation fluence, and $\Delta\omega$ normalised by the carrier density $N_{carrier}$ that gives rise to the Drude response. The values are plotted as a function of $N_{carrier}$, which was also extracted from the fits to the spectra and is equivalent to the average carrier density. The fitted $N_{carrier}$ was chosen for the plot to account for slight variations from predicted photoexcitation density due to errors in the measured laser power and saturation effects at higher fluence. We note, however, that using values directly calculated from the measured power of the pump provides virtually the same result.

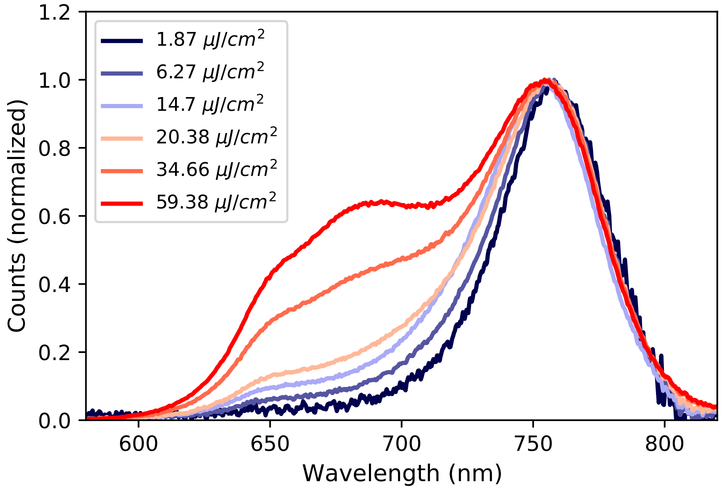

Supplementary Figure 10 PL spectra of the $MAPb(I_{0.5}Br_{0.5})_3$ film after phase segregation, taken with 400 nm pulsed photoexcitation at increasing fluences.

Supplementary Note 2. Charge carrier dynamics

The $\Delta T/T$ dynamics can be related to the decay of the charge-carrier density over time $n(t)$. The transients were modelled as described elsewhere[4] with the rate equation

$$\frac{dn}{dt} = -k_1 n - k_2 n^2 - k_3 n^3 \tag{11}$$

where k_1, k_2 and k_3 are the first-, second-, and third-order recombination rate constants, respectively. We consider the contribution of excitons to be negligible, based on the low exciton binding energies in these materials.[5]

k_1 was extracted from the PL transients, which are acquired at lower fluence and longer timescale. k_3 has a minimal contribution at the excitation densities employed so it was manually adjusted and then fixed to 1×10^{-28} cm^6s^{-1}. The OPTP dataset with various fluences was globally fitted, allowing k_2 to vary. When using Equation 11 for fitting the OPTP transients, μ was considered to be constant. This is a fair approximation in this case, as we have demonstrated that μ is similar in the majority and I-rich phases. The adapted model with funnelling dynamics takes into account the distinct μ for each phase in the halide-segregated film (see section B of this Note).

A. Diffusion and photon reabsorption

To account for the varying photogeneration density through the thickness of the film, the fit routine takes into account the exponential charge-density profile created by the excitation light by dividing the sample into 150 slices of equal thickness. The decay is computed individually for each slice and then added

together to obtain the final modelled OPTP decay.

Carriers are allowed to diffuse between slices across the film depth according to the diffusion coefficient

$$D = \frac{\mu k_b T}{e} \tag{12}$$

where μ is the charge-carrier mobility, k_b is the Boltzmann constant, T is the temperature and e is the elementary charge. Diffusion is implemented using a forward Euler method taking the gradient of carrier distribution at intervals dt_{diff}, which is determined based on the calculation parameters to allow for stable solution. Recombination is calculated by Equation 11 at intervals of $dt_{recombination} = 10 \times dt_{diff}$ The absorption and emission spectra of the film are used to calculate the profile of reabsorption across the film for photons emitted by each film slice at any time step (given by $k_2 n^2$). The internal reflections of emitted photons at both film interfaces is calculated by transfer matrix methods, and the reabsorption is calculated for emitted photons propagating a total distance of $L_{PL} = 2 \times D$, where D is the film thickness. This was found to be a fair approximation for the interface reflectivities in the samples studied here.

B. Funnelling dynamics

The fast occurrence of charge funnelling in the phase-segregated mixed halide film is indicated by the time-resolved PL spectra (Figure 11), where the absence of a high-energy component or a slow rise of the low-energy emission suggest that funneling is faster than the experiment resolution of $\sim$1 ns. This is supported by the observation of fast changes to the phonon features associated with the segregated I-rich domains in the time-resolved OPTP photoconductivity spectra (Figure 12). Based on these observations, we modelled the charge funnelling dynamics to evaluate its impact on the charge-carrier recombination.

The charge-carrier dynamics in the phase-segregated $MAPb(I_{0.5}Br_{0.5})_3$ film was modelled by taking into account the presence of two phases: one majority phase and one I-rich phase taking a fraction R_{vol} of the film volume. For simplicity, R_{vol} is considered to be constant across the film depth. Upon 400 nm photoexcitation, charge-carriers are generated into each phase with equal densities. As summarized by Equations 13,14, carriers within the majority phase (n_M) undergo recombination according to $k_{M,1}$, $k_{M,2}$, and $k_{M,3}$, or can be transferred to the I-rich phase with a first order rate constant $k_{transfer}$. Carriers within the I-rich phase (n_I) recombine according to $k_{I,1}$, $k_{I,2}$, and $k_{I,3}$.

$$\frac{dn_M}{dt} = -k_{M,1} n_M - k_{M,2} n_M^2 - k_{M,3} n_M^3 - k_{transfer} n_M + k_{back}(n_I/n_{max}) n_I \tag{13}$$

$$\frac{dn_I}{dt} = -k_{I,1} n_I - k_{I,2} n_I^2 - k_{I,3} n_I^3 + k_{transfer} n_M - k_{back}(n_I/n_{max}) n_I \tag{14}$$

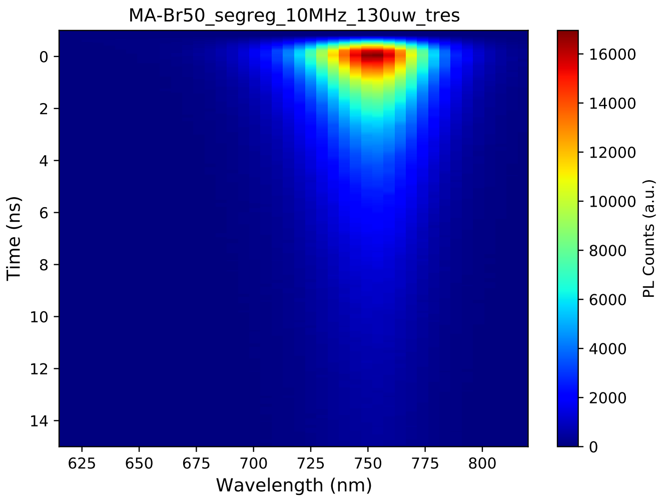

Supplementary Figure 11 Spectrally resolved PL decay of the phase-segregated $MAPb(I_{0.5}Br_{0.5})_3$ film under 400 nm photoexcitation at 50 nJ/cm^2.

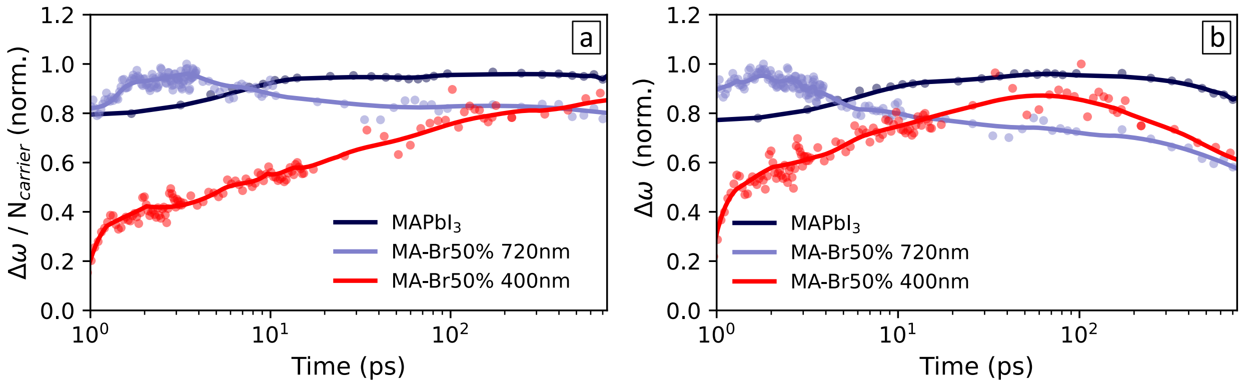

Supplementary Figure 12 (a) Photoexcitation induced phonon frequency shift $\Delta\omega$ divided by carrier density N (and subsequently normalized for comparison), extracted from fits to the photoconductivity spectra as a function of time delay following photoexcitation. Dots show the fitted values extracted from data of a $MAPbI_3$ thin film with 400 nm photoexcitation (dark blue), $MAPb(I_{0.5}Br_{0.5})_3$ thin film with 400 nm (red) and 720 nm pump (light blue), respectively. Solid lines are splines to guide the eye. The division by N in each curve was performed to remove variations associated with recombination losses over time delay. (b) shows the (normalized) values of $\Delta\omega$ without division by N.

A back-transfer rate k_{back} is implemented to account for the saturation of the I-rich domains. To better describe the effect of band-filling, k_{back} is regulated according to the density of charge-carriers within the I-rich phase and a maximum density n_{max}. Such a parameter relates to the maximum available states in the I-rich phase below the bandgap of the majority phase, and depends on the band alignment and the Fermi distribution of carriers. Because we are unable to accurately determine n_{max}, its value is adjusted to the experimental data in such a way as to reproduce the saturation effect observed in the PL spectra with increasing photoexcitation intensities (Figure 10). $k_{transfer}$ is kept as a fixed first order rate constant for simplicity, which is a valid assumption based on the fact that the density of I-rich domains throughout the film remains constant regardless of the carrier population within each domain.

Furthermore, carrier diffusion takes place within each phase (as described in section A of this Note). We note however, that owing to the high carrier concentration in the I-rich phase as a result of funnelling, recombination occurs very fast and thus we did not observe a very significant contribution of carrier diffusion within the I-rich phase.

The photon reabsorption effect is calculated based on four scenarios: photons emitted by the majority phase and captured by the majority phase ($P_M A_M$), or captured by the I-rich phase ($P_M A_I$), and photons emitted by the I-rich phase and captured by the majority phase ($P_I A_M$), or captured by the I-rich phase ($P_I A_I$). The reabsorption probability profile is calculated for each of these four conditions based on the absorption and emission spectra, and used to calculate the profile of reabsorbed photons within each phase at any point in time.

Because the photons emitted by the majority phase are more efficiently recaptured in the film, the fraction of photon recycling calculated by the model depends on the intensity of excitation, and can vary from $\sim$15% for emission mainly from the I-rich phase to $\sim$50% at 60 μJ/cm^2.

The contribution of carriers to the OPTP $\Delta T/T$ signal in each phase is proportional to the experimentally measured mobilities ($\mu_{major} = 37$ and $\mu_{I-rich} = 49$ cm^2/(Vs)).

We consider $R_{vol} = 1\%$ based on previous studies.[11,12] We fixed $k_{M,1} = k_{I,1}$ and $k_{M,3} = k_{I,3}$, while $k_{M,2}$ was fixed to the fitted k_2 for the mixed halide film before phase segregation and $k_{I,2}$ was fixed to the value obtained from fits to data for direct photoexcitation into the I-rich phase. The initially estimated values of $k_{transfer}$ and k_{back} were chosen to be in close agreement with the fast funnelling observed in the THz photoconductivity spectra as a function of pump time delay t_{pump} (Figure 3 of the main text), and to be able to reproduce the saturation of I-rich domains observed in the fluence dependence of the PL spectra (Figure 10). From these initial estimates, $k_{transfer}$ and k_{back} were then fitted to the experimental OPTP transients. Table 1 summarizes the values obtained from fits to experimental data and the values adopted for modelling the dynamics. The boundaries for k_2 and μ in the I-rich phase (accounting to uncertainties

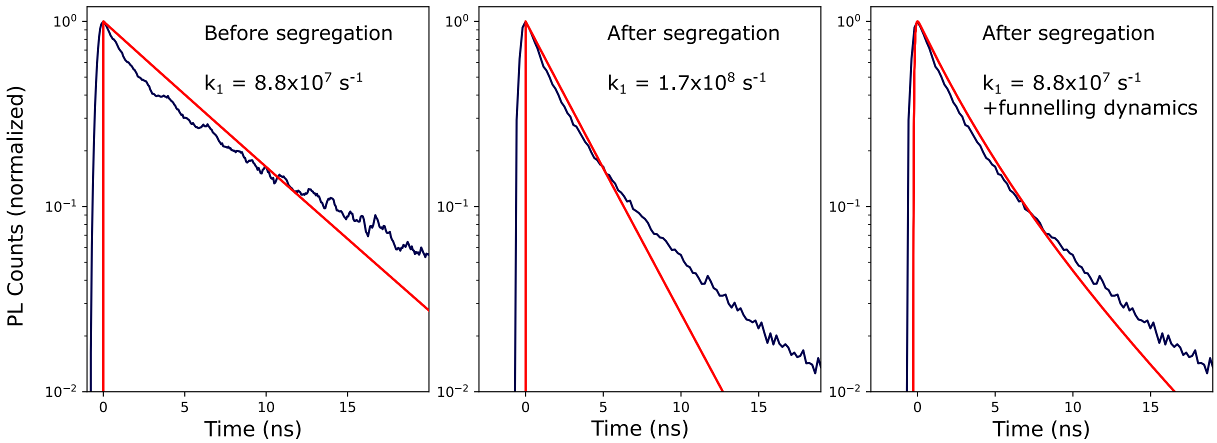

Supplementary Figure 13 Experimental PL dynamics (blue) before and after segregation, and simulated PL dynamics extracted from our model either with increased fitted values of k_1 or maintaining the same values of k_1 but accounting for charge-funneling dynamics.

in charge-carrier population) are $k_{2(lower)} = 1.9 \times 10^{-9}$ cm^{-3}s^{-1}, $k_{2(upper)} = 6 \times 10^{-9}$ cm^{-3}s^{-1}, $\mu_{lower} = 35$ cm^2/(Vs), and $\mu_{upper} = 66$ cm^2/(Vs) (see Supplementary Note 6 for details).

Supplementary Table 1 Recombination rate constants. Values marked by * have not been directly obtained from experimental data but assumed based on the observations and adopted for modelling the recombination dynamics. For simplicity, k_3 was fixed to 1×10^{-28} cm^{6}s^{-1} for all fits and simulations. The lower and upper boundaries (accounting for uncertainties in the calculation of charge-carrier dynamics) for μ and k_2 in the I-rich phase have been detailed in Supplementary Note 6.

Material	μ (cm^{2}V^{-1}s^{-1})	k_1 (10^6 s^{-1})	k_2 (10^{-9} cm^{3}s^{-1})	k_3 (10^{-28} cm^{6}s^{-1})
Mixed phase (before segregation)	37.3±2.7	88±2	2.7±0.1	1<br>1
Majority phase	37.2±0.6	88*	2.7*	1
I-rich phase	49 (35< μ <66)	88*	2.5 (1.9< μ <6)	1
Phase-segregated (effective values)	37.2±0.6	170±10	4.6±0.2	1

The values of $k_{M,1}$ and $k_{M,2}$ were taken from the fits to the 400 nm photoexcitation OPTP data before phase segregation. The best value found for $k_{transfer}$ was 9 $\times 10^9$ s^{-1}. The adapted model can reproduce the PL dynamics after phase segregation without changing the initial fitted k_1 value (Figure 13). We note that photoinduced ion migration is known to have dramatic effects, both positive and negative, on the density of charge-trapping defects in perovskites,[13,14] thus it is very likely that the trap-assisted recombination rate constant k_1 is not fully preserved following phase segregation. It is also important to note that the model described here does not account for the heterogeneous distribution of I-rich domains through the phase-segregated films, which are likely to be concentrated at grain boundaries and at the illuminated surface of the film. The model also considers only two distinct phases rather than a more complex and realistic case where various gradual halide compositions can coexist in the material. These

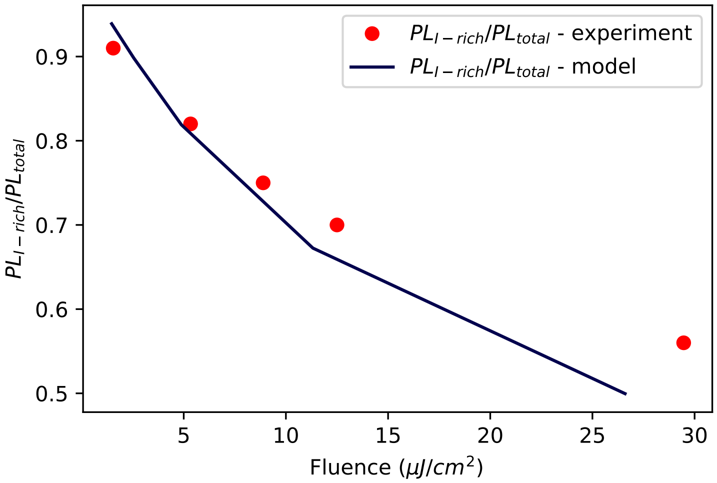

Supplementary Figure 14 Ratio of PL emission originating from the I-rich domains as a function of photoexcitation fluence. Red dots are experimental values (from spectra in Figure 10) and line is extracted from the model.

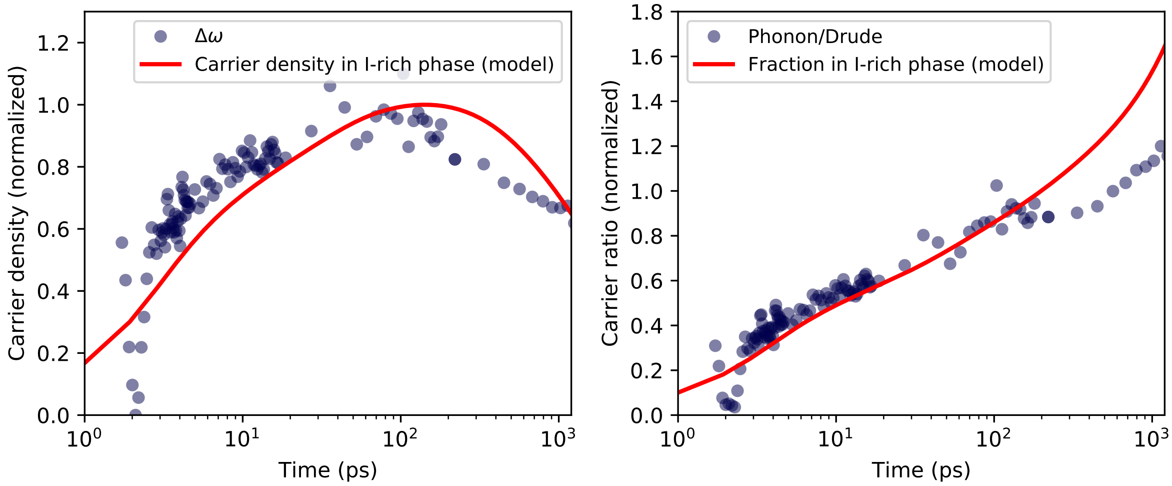

Supplementary Figure 15 Increase of carrier density and fraction of total carrier population within I-rich domains given by the charge funnelling model (red), compared with the phonon shifts *Delta*ω and the ratio between Phonon/Drude contributions extracted from the fitted OPTP photoconductivity spectra over pump time delay.

factors contribute to more complex dynamics that cannot be fully described by the model presented here. However, our model can reproduce with a good level of agreement a series of observations that include the OPTP transients (Figure 4a in the main text), the change in PL lifetimes (Figure 4b in the main text), and the effect of saturation of the I-rich phase on the PL spectra with increasing illumination intensities (Figures 10 and 14), while being consistent with the dynamic changes observed in the THz photoconductivity spectral features (Figure 15).

Although we cannot at this stage accurately quantify how the magnitude of phonon shift $\Delta\omega$ scales with charge-carrier densities, we can observe an overall agreement between the values of $\Delta\omega$ fitted to the photoconductivity spectra and the carrier densities within the I-rich phase extracted from the dynamic model (Figure 15).

Supplementary Note 3. The role of charge-carrier concentration in the enhancement of phonon anharmonicity

Finally, we assess the impact of increased charge-carrier concentration on the observation of enhanced phonon anharmonicity. For this purpose, we re-evaluate the photoexcitation density dependence of $\Delta\omega$ (Figure 9) accounting for the heterogeneous charge-carrier densities extracted from our model. Although the average carrier density is directly related to the amplitude of the Drude contribution to the photoconductivity spectra, the concentration of carriers in certain regions can result in a stronger effect of lattice anharmonicity experienced by the charges giving rise to the photoconductivity. Too account for this effect, we define a weighted charge-carrier density N^* given by:

$$N^* = R_N N_{I-rich} + (1 - R_N) N_{majority} \tag{15}$$

where R_N is the fraction of total carriers concentrated in the I-rich phase.

The values of N^* at various photoexcitation fluences were calculated from the simulated populations decays. We then analyse the relation between N^* and $\Delta\omega$ fitted to experimentally acquired photoconductivity spectra of the phase-segregated $MAPb(I_{0.5}Br_{0.5})_3$ film with 400 nm photoexcitation. The values of $\Delta\omega$ and $\Delta\omega/N^*$ as a function of N^* are plotted in Figure 16 in light blue. For reference we also show the values obtained for $MAPbI_3$ and $MAPb(I_{0.5}Br_{0.5})_3$, where the average density N extracted from the fitted Drude contribution was used instead of N^* (as previously shown in Figure 9).

We can observe that when accounting for the variations in charge-carrier densities that result from charge funnelling, the magnitude of phonon anharmonicity in the phase segregated mixed halide perovskite appears to behave similarly to that for a $MAPbI_3$ film.

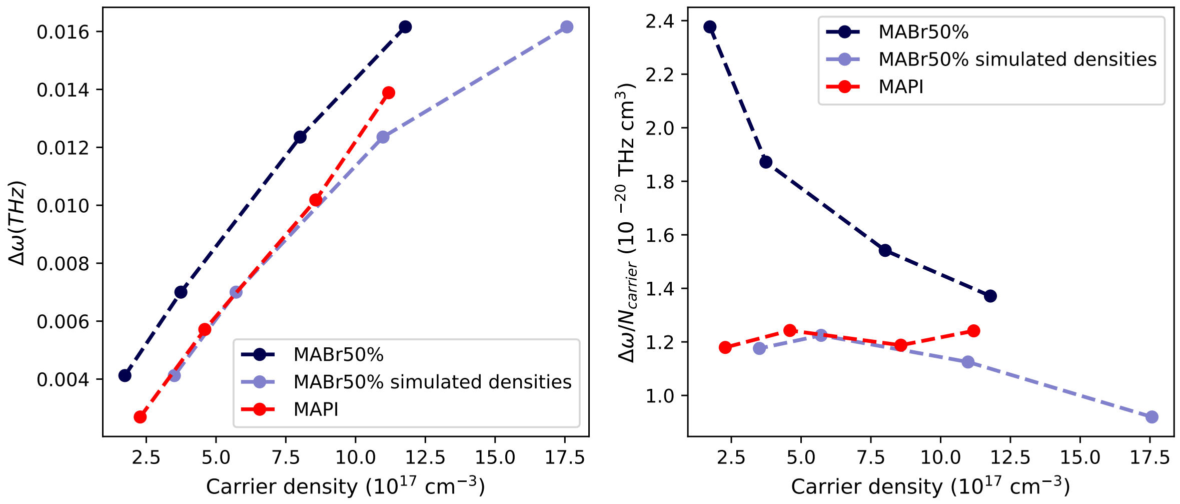

Supplementary Figure 16 Phonon shift $\Delta\omega$ extracted from the THz photoconductivity spectra of phase-segregated $MAPb(I_{0.5}Br_{0.5})_3$ and $MAPbI_3$ with 400 nm photoexcitation with increasing photoexcitation fluence, and $\Delta\omega$ normalised by the carrier density $N_{carrier}$ and plotted as a function of $N_{carrier}$. In dark blue ($MAPb(I_{0.5}Br_{0.5})_3$) and red ($MAPbI_3$), $N_{carrier}$ is extracted from the fitted Drude contribution to the spectra. In light blue we show the values for $MAPb(I_{0.5}Br_{0.5})_3$ but instead of $N_{carrier}$ we use the balanced carrier density N^* extracted from the simulated populations.

Although the photoconductivity spectra were acquired at pump time delay $t_{pump} = 5$ ps, we adopted the values of N^* at the point of highest density within the I-rich phase. This was deliberately done because as seen in Figure 15, our model does not necessarily give a precise account of the populations at such early timescales. However, given the success of our model in reproducing the enhanced radiative rates associated with the increased densities within the I-rich phase, this analysis demonstrates that such carrier concentration can also be a substantial factor behind the enhancement of phonon anharmonicity in phase-segregated mixed halide perovskites.[15]

One possible conclusion to be drawn from this observation is that the phase-segregated domains in the mixed halide film does not necessarily present stronger phonon anharmonicity with respect to a phase-pure I-rich perovskite lattice.

Supplementary Note 4. The effects of background illumination on OPTP observations

To confirm that the changes observed to the THz photoconductivity spectra and dynamics in the mixed halide perovskite film were associated with phase segregation and not degradation or any other effect of the CW background illumination, we performed the experiments with $MAPbI_3$ films for reference. Figure 17 and 18 shows the THz photoconductivity spectra and the OPTP transients, respectively, for a spin-

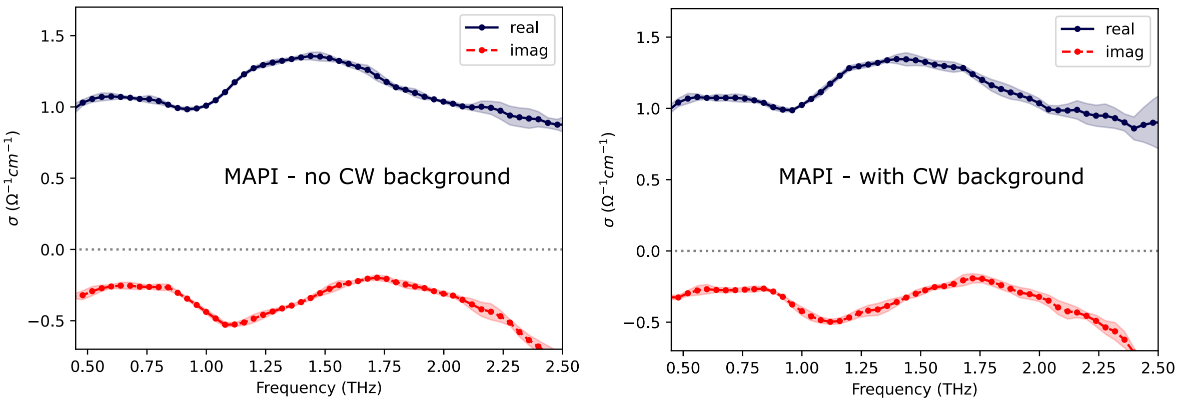

Supplementary Figure 17 THz photoconductivity spectra of $MAPbI_3$ with 400 nm photoexcitation at fluence 20 μJ/cm^2, with and without background illumination with a 532 nm CW laser (∼500 mW/cm^2).

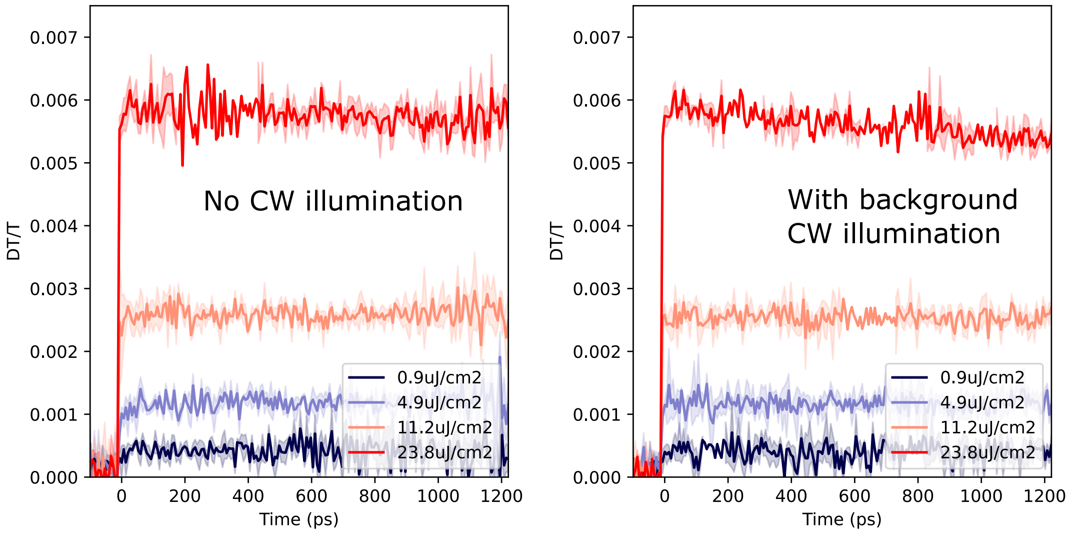

Supplementary Figure 18 THz photoconductivity dynamics of a spin-coated $MAPbI_3$ film with 400 nm photoexcitation, with and without background illumination with a 532 nm CW laser (∼100 mW/cm^2).

coated $MAPbI_3$ film before any exposure to the 532 nm CW laser and after/during continuous exposure. No significant variations were observed. The experiment was also performed on a vapour-deposited $MAPbI_3$ film that is characterised by shorter charge-carrier lifetimes. Again, no significant impact of the CW illumination was observed.

Photoinduced ion migration is known to have dramatic effects, both positive and negative, on the density of charge-trapping defects in perovskites,[13,14] thus it is likely that the trap-assisted recombination rate constant k_1 is not fully preserved following phase segregation. This is minimised in our experimental conditions because of the low CW illumination intensity, large illumination areas and the encapsulation of the perovskite film with a PMMA layer.

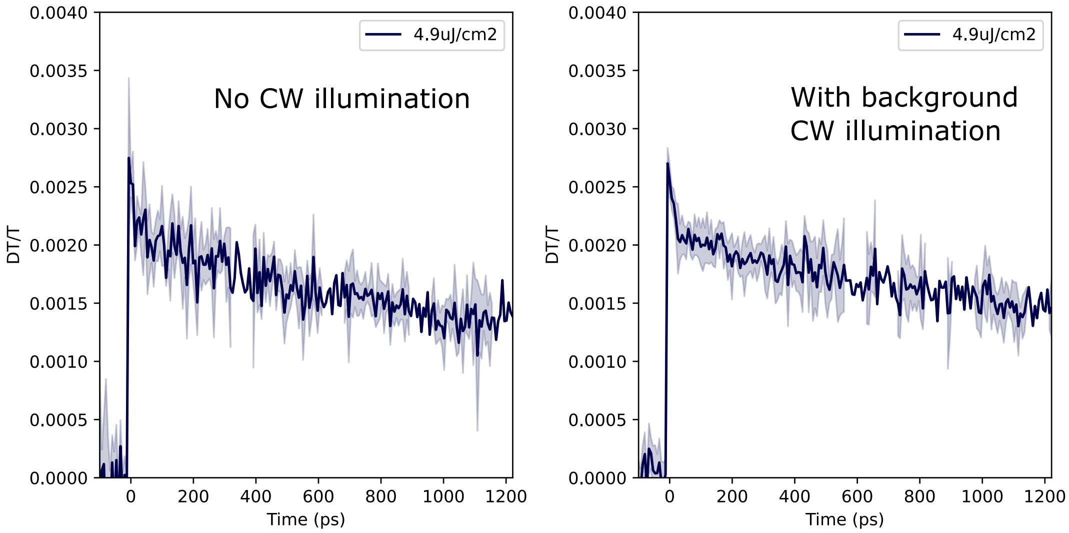

Supplementary Figure 19 THz photoconductivity dynamics of a vapour-deposited $MAPbI_3$ film with 400 nm photoexcitation, with and without background illumination with a 532 nm CW laser ($\sim$100 mW/cm^2).

Supplementary Note 5. Reversibility of halide segregation

Experiments have been performed on the sample after recovery in dark to confirm the reversibility of the halide segregation and the effects observed in the OPTP dynamics and spectra. Figure 20 shows the recovery in phonon anharmonicity effects observed in the photoconductivity spectrum, and Figure 21 shows the recovery in lifetime shortening.

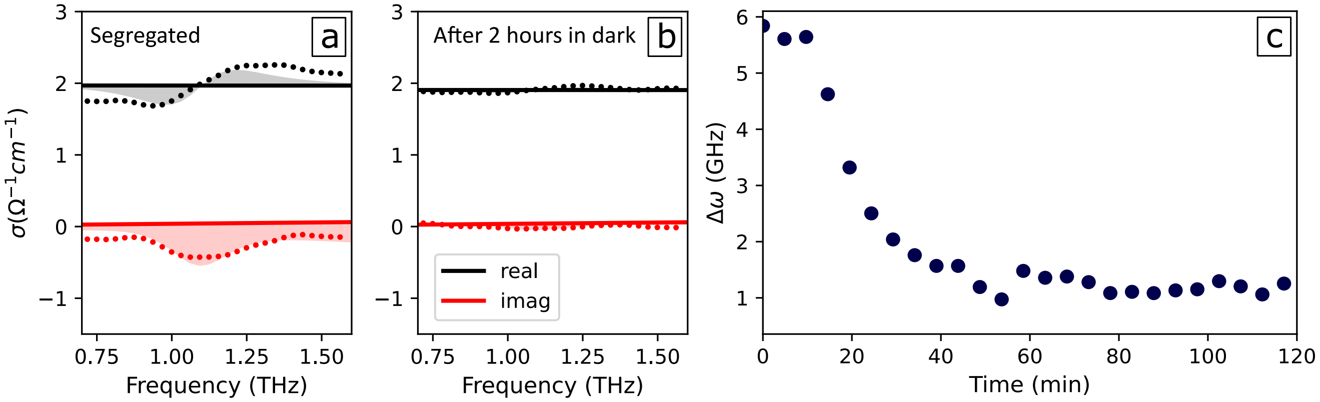

Supplementary Figure 20 OPTP photoconductivity spectra of $MAPb(I_{0.5}Br_{0.5})_3$ thin film after phase segregation (a) and after two hours of recovery without the CW illumination (b). (c) Values of $\Delta\omega$ obtained from fits to the OPTP spectra after the CW illumination was blocked.

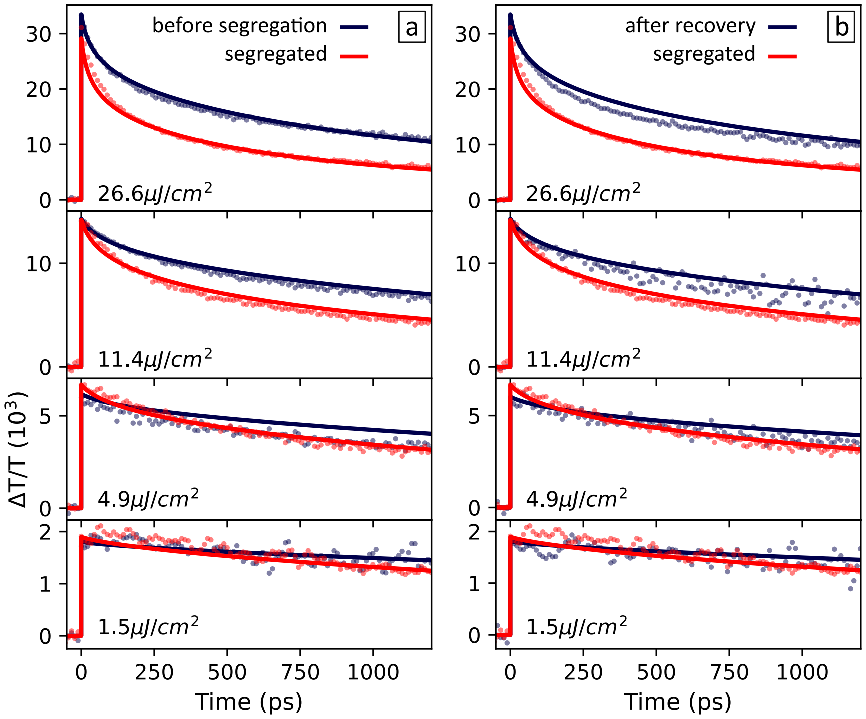

Supplementary Figure 21 (a) OPTP transients of $MAPb(I_{0.5}Br_{0.5})_3$ thin film at various photoexcitation fluences, before (dark blue) and after (red) phase segregation. (b) OPTP transients after (red) phase segregation compared to the transients acquired after four hours of recovery in dark. Dots are experimental data and solid lines are fits to the model described in Supplementary Note 2. The dark blue solid lines in both panels equally show the fitted curves of the mixed film before segregation to allow for a better comparison of the dynamics before and after recovery.

Supplementary Note 6. Calculation of charge-carrier population upon direct photoexcitation of I-rich domains

The calculation of THz effective charge mobilities depends on the determination of charge-carrier population generated by the photons absorbed from the photoexcitation pulse. To account for this effect, we measure the absorption coefficients of the sample and use transfer matrix methods to calculate the profile of photon absorption across the semiconductor film. We consider one charge-carrier generated for each absorbed photon. For high energy photoexcitation such as 400 nm light, the optical density of the perovskite is normally high enough that virtually all of the incident light that is not reflected is absorbed by the film. For the 720 nm photoexcitation into the I-rich domains, however, the optical density is much lower and hence the calculation of charge-carrier population carries more uncertainties. To confirm that the low energy pump did not generate charge-carriers in the mixed-halide majority phase, Figure 22 shows negligible THz signal using 700 and 720 nm pump before halide segregation occurs.

The absorption spectrum of the $MAPb(I_{0.5}Br_{0.5})_3$ film was measured (following the procedure described

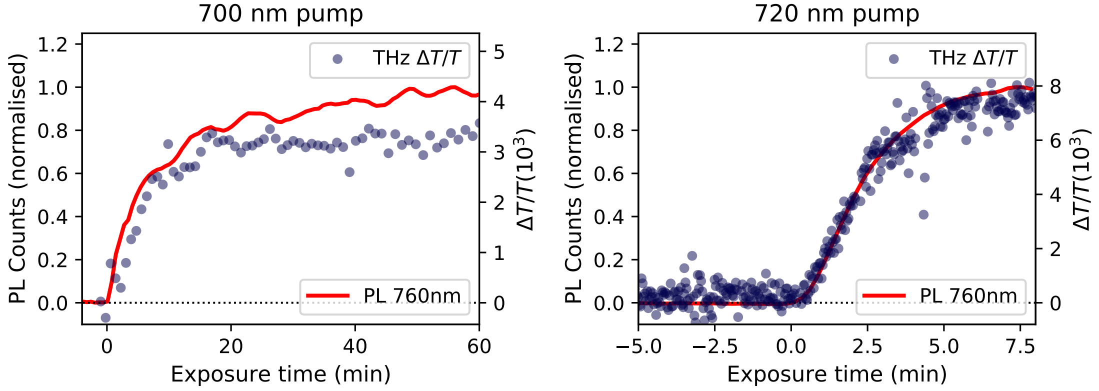

Supplementary Figure 22 OPTP $\Delta T/T$ signal under 700 and 720 nm photoexcitation and PL signal at 760 nm measured simultaneously. At $t_{exposure} = 0$, phase segregation is induced with the 532 nm CW laser.

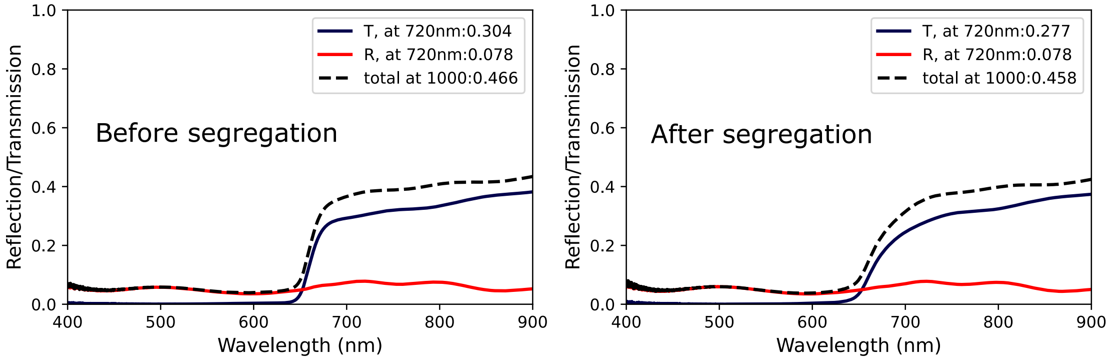

Supplementary Figure 23 Transmission and reflection of the $MAPb(I_{0.5}Br_{0.5})_3$ film before and after halide segregation. Scattering background has been subtracted.

in Supplementary Methods) before and immediately after halide segregation induced by exposure to the 532 nm CW laser. Before halide segregation the band-edge of the semiconductor is sharp, with a well defined exciton peak. Phase segregation results in a broadened band-edge and increased absorption at wavelengths above 650 nm (Figure 24). In accordance with halide segregation occurring only in a minority portion of the semiconductor volume,[11,12] no significant shifts of the bandgap are discernible in the absorption spectra. The accurate evaluation of the optical densities at these long wavelengths suffer from interference artifacts and from the high level of light scatter by sample inhomogeneities. This is highlighted by the sum of transmittance (T) and reflectance (R) values below the bandgap, which falls short of the ideal value of 1 (Figure 23).

Method 1

As a first approach to calculate the charge-carrier population generated by the 720 nm pump into the I-rich domains of the phase-segregated perovskite we removed the scattering background from the absorption

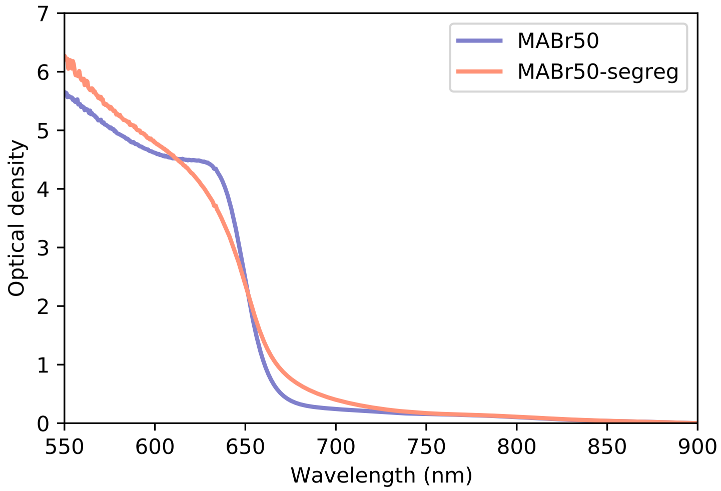

Supplementary Figure 24 Absorption spectra of the $MAPb(I_{0.5}Br_{0.5})_3$ film before and after halide segregation. Scattering background has been subtracted. Note that the bandgap is mostly unchanged after phase segregation, owing to the occurrence of halide segregation in only a small portion of the material's total volume.

spectrum by subtracting the value below the bandgap at 850 nm. The optical densities obtained from the background-subtracted spectrum after phase segregation leads to a calculated THz mobility of 66 $cm^2/(Vs)$. We note, however, that due to the high degree of scattering in the sample, this background correction is somewhat accurate, particularly in the case of the phase-segregated film, where the bandedge is broader and there are more uncertainties in the subgap absorption. As the fraction of the film occupied by I-rich domains is expected to be low, the real absorption of the film at 720 nm is likely to be hidden by the scattering background which is subtracted. The charge-carrier populations calculated by this method are therefore relatively low, resulting in such high values for mobility.

Method 2

We also estimate the lowest boundary for mobility using the absorption coefficient measured for $MAPbI_3$ and calculating the optical density for the extreme case of the I-rich phase occupying 50% of the film volume. The charge-carrier population calculated from this method results in a mobility value of 35 $cm^2/(Vs)$.

Method 3

Finally, as an alternative approach, we evaluate separately the changes in transmission and reflection of the photoexcitation light. We observe from the data in Figure 23 that the fraction of reflected 720 nm light R_{720} is very similar before and after halide segregation. This is related to the presence of the protecting PMMA layer over the perovskite film and also in agreement with the occurrence of halide segregation in

a limited volume of the semiconductor, which is not expected to greatly affect the overall film reflectance. Furthermore, these values match the R_{720} values obtained from transfer matrix methods. Therefore, we consider R_{720} to be constant and equal to 0.078.

We then analyse the impact of phase segregation on T_{720}. Due to scattering and interference artifacts, the transmission measured by the methods described in (23) cannot be taken with a high degree of confidence. So we compare three other different methods for the determination of T_{720}. First we calculated T_{720} from the background-corrected optical density (Figure 24), from which we obtain a decrease in transmission of ~ 0.06. Second, we directly measured the 720 nm laser power immediately after the sample mounted in the OPTP setup before and after phase segregation occurs, which gives us $T_{720} = 0.4$ before segregation and $T_{720} = 0.3$ after segregation. In this approach the path between the sample and the power measurement is shorter in comparison to the spectrometer measurement described in , resulting in less scattering losses.

As a final comparison, we estimated the expected optical density of a phase-segregated film containing 1% I-rich perovskite phase. This was obtained from the linear combination of the background-corrected absorption coefficients of $MAPb(I_{0.5}Br_{0.5})_3$ before phase segregation multiplied by a scaling factor of 0.99 and the absorption coefficients measured for $MAPbI_3$ multiplied by a scaling factor of 0.1. We note that this approach is not completely accurate as it does not consider the broad distribution of bandgaps in the film[11] or the fact that the I-rich phase formed upon halide segregation is not entirely comprised of $MAPbI_3$ perovskite, but rather a low non-zero Br composition.[16] However, the optical densities obtained from this approach also predict a decrease in T_{720} of ~ 0.1, which is very consistent with the two first methods described above.

So we establish that $T_{720} = T_{720-mixed} + 0.1$, where $T_{720-mixed}$ is the transmittance before phase segregation. Despite the negligible photoconductivity signal before phase segregation (Figure 22), we do not entirely dismiss any absorption of 720 nm light in the non-segregated film. Some subgap absorption can be associated with subgap states that would not result in photoconductivity signal. Instead we calculated $T_{720-mixed}$ with transfer matrix methods from the background-corrected absorption spectrum of the mixed phase film before segregation.

From the determined values of T_{720} and R_{720} we calculate 49 $cm^2/(Vs)$ and the mobility of the charge-carriers generated by the 720 nm photoexcitation.

We take $\mu = 49$ $cm^2/(Vs)$ as our best approximation for the THz effective mobility of the I-rich phase, with lower and upper boundaries of $\mu_{lower} = 35$ $cm^2/(Vs)$ and $\mu_{upper} = 66$ $cm^2/(Vs)$ based on our different methods for the calculation.

Effect on the recombination rates

The determination of recombination rate constants also depend on the calculation of carrier densities. The fitted value of k_2 obtained by method 1 of calculating the carrier population is 6×10^{-9} cm^{3}s^{-1}. By applying method 2, we obtain a value of $k_2 = 1.9 \times 10^{-9}$ cm^{-3}s^{-1}, and method 3 yields $k_2 = 2.5 \times 10^{-9}$ cm^{-3}s^{-1}.

Analogously to the determination of charge-carrier mobilities, we adopt the value of $k_2 = 2.5 \times 10^{-9}$ cm^{-3}s^{-1} as our best estimation, and to account for the uncertainties we establish lower and upper boundaries of $k_{2(lower} = 1.9 \times 10^{-9}$ cm^{-3}s^{-1} and $k_{2(upper} = 6 \times 10^{-9}$ cm^{-3}s^{-1}.

This upper boundary of k_2 is over twice as high as the k_2 obtained for the majority phase. We note, however, that the calculation method that yields the upper value of k_2 also yields the upper values mobility μ = 66 cm^2/(Vs). While higher bimolecular rates can result from confinements effects, such high mobilities would present a strong contradiction to the existence of confinement. Such inconsistencies in the calculated upper limits for k_2 and μ are indicative of the underestimation of charge-carrier densities using method 1, and serve as further support that the actual values are safely placed within the boundaries we have established.

Supplementary References

[1] N. K. Noel, S. N. Habisreutinger, B. Wenger, M. T. Klug, M. T. Hörantner, M. B. Johnston, R. J. Nicholas, D. T. Moore, and H. J. Snaith, "A low viscosity, low boiling point, clean solvent system for the rapid crystallisation of highly specular perovskite films," *Energy & Environmental Science*, vol. 10, no. 1, pp. 145–152, 2017.

[2] A. J. Knight, A. D. Wright, J. B. Patel, D. P. McMeekin, H. J. Snaith, M. B. Johnston, and L. M. Herz, "Electronic Traps and Phase Segregation in Lead Mixed-Halide Perovskite," *ACS Energy Letters*, vol. 4, pp. 75–84, jan 2019.

[3] A. J. Knight, J. Borchert, R. D. J. Oliver, J. B. Patel, P. G. Radaelli, H. J. Snaith, M. B. Johnston, and L. M. Herz, "Halide Segregation in Mixed-Halide Perovskites: Influence of A-Site Cations," *ACS Energy Letters*, vol. 6, pp. 799–808, feb 2021.

[4] C. Wehrenfennig, G. E. Eperon, M. B. Johnston, H. J. Snaith, and L. M. Herz, "High Charge Carrier Mobilities and Lifetimes in Organolead Trihalide Perovskites," *Advanced Materials*, vol. 26, pp. 1584–1589, mar 2014.

[5] V. D'Innocenzo, G. Grancini, M. J. P. Alcocer, A. R. S. Kandada, S. D. Stranks, M. M. Lee, G. Lanzani, H. J. Snaith, and A. Petrozza, "Excitons versus free charges in organo-lead tri-halide perovskites.," *Nature Communications*, vol. 5, p. 3586, jan 2014.

[6] "Direct measurement of the exciton binding energy and effective masses for charge carriers in organic–inorganic tri-halide perovskites," *Nature Physics*, vol. 11, pp. 582–587, jul 2015.

[7] C. La-o vorakiat, L. Cheng, T. Salim, R. A. Marcus, M.-E. Michel-Beyerle, Y. M. Lam, and E. E. M. Chia, "Phonon features in terahertz photoconductivity spectra due to data analysis artifact: A case study on organometallic halide perovskites," *Applied Physics Letters*, vol. 110, p. 123901, mar 2017.

[8] A. M. Ulatowski, L. M. Herz, and M. B. Johnston, "Terahertz Conductivity Analysis for Highly Doped Thin-Film Semiconductors," *Journal of Infrared, Millimeter, and Terahertz Waves*, vol. 41, pp. 1431–1449, dec 2020.

[9] D. Zhao, H. Hu, R. Haselsberger, R. A. Marcus, M.-E. Michel-Beyerle, Y. M. Lam, J.-X. Zhu, C. La-o vorakiat, M. C. Beard, and E. E. M. Chia, "Monitoring Electron–Phonon Interactions in Lead Halide Perovskites Using Time-Resolved THz Spectroscopy," *ACS Nano*, vol. 13, pp. 8826–8835, aug 2019.

[10] K. T. Munson, J. R. Swartzfager, and J. B. Asbury, "Lattice Anharmonicity: A Double-Edged Sword for 3D Perovskite-Based Optoelectronics," *ACS Energy Letters*, vol. 4, pp. 1888–1897, aug 2019.

[11] S. Mahesh, J. M. Ball, R. D. J. Oliver, D. P. McMeekin, P. K. Nayak, M. B. Johnston, and H. J. Snaith, "Revealing the origin of voltage loss in mixed-halide perovskite solar cells," *Energy & Environmental Science*, vol. 13, pp. 258–267, jan 2020.

[12] E. T. Hoke, D. J. Slotcavage, E. R. Dohner, A. R. Bowring, H. I. Karunadasa, and M. D. McGehee, "Reversible photo-induced trap formation in mixed-halide hybrid perovskites for photovoltaics," *Chem. Sci.*, vol. 6, pp. 613–617, nov 2014.

[13] S. G. Motti, D. Meggiolaro, S. Martani, R. Sorrentino, A. J. Barker, F. De Angelis, and A. Petrozza, "Defect Activity in Lead Halide Perovskites," *Advanced Materials*, vol. 31, p. 1901183, nov 2019.

[14] S. G. Motti, D. Meggiolaro, A. J. Barker, E. Mosconi, C. A. R. Perini, J. M. Ball, M. Gandini, M. Kim, F. De Angelis, and A. Petrozza, "Controlling competing photochemical reactions stabilizes perovskite solar cells," *Nature Photonics*, vol. 13, pp. 532–539, aug 2019.

[15] C. Q. Xia, S. Poncé, J. Peng, A. M. Ulatowski, J. B. Patel, A. D. Wright, R. L. Milot, H. Kraus, Q. Lin, L. M. Herz, F. Giustino, and M. B. Johnston, "Ultrafast photo-induced phonon hardening due to Pauli blocking in MAPbI 3 single-crystal and polycrystalline perovskites," *Journal of Physics: Materials*, vol. 4, p. 044017, oct 2021.

[16] A. J. Barker, A. Sadhanala, F. Deschler, M. Gandini, S. P. Senanayak, P. M. Pearce, E. Mosconi, A. J. Pearson, Y. Wu, A. R. Srimath Kandada, T. Leijtens, F. De Angelis, S. E. Dutton, A. Petrozza, and R. H. Friend, "Defect-Assisted Photoinduced Halide Segregation in Mixed-Halide Perovskite Thin Films," *ACS Energy Letters*, vol. 2, pp. 1416–1424, jun 2017.
